# Rapid Phenotypic and Genotypic Antimicrobial Susceptibility Testing Approaches for Use in the Clinical Laboratory

**DOI:** 10.3390/antibiotics13080786

**Published:** 2024-08-22

**Authors:** Siham Hattab, Adrienne H. Ma, Zoon Tariq, Ilianne Vega Prado, Ian Drobish, Rachel Lee, Rebecca Yee

**Affiliations:** 1 Department of Pathology, George Washington University School of Medicine and Health Sciences, Washington, DC 20037, USA; shattab@mfa.gwu.edu (S.H.); ztariq@mfa.gwu.edu (Z.T.); ilprado@mfa.gwu.edu (I.V.P.); 2 Department of Pharmacy, Valley View Hospital, Glenwood Springs, CO 81647, USA; adrienne.ma@vvh.org; 3 Critical Care Medicine Department, National Institutes of Health, Bethesda, MD 20892, USA; ian.drobish@nih.gov; 4 Division of Infectious Diseases, George Washington University School of Medicine and Health Sciences, Washington, DC 20037, USA; lee.rachelraven@gmail.com

**Keywords:** rapid antimicrobial susceptibility, antimicrobial resistance, infection prevention, diagnostics, molecular technologies, multiplex PCR

## Abstract

The rapid rise in increasingly resistant bacteria has become a major threat to public health. Antimicrobial susceptibility testing (AST) is crucial in guiding appropriate therapeutic decisions and infection prevention practices for patient care. However, conventional culture-based AST methods are time-consuming and labor-intensive. Therefore, rapid AST approaches exist to address the delayed gap in time to actionable results. There are two main types of rapid AST technologies— phenotypic and genotypic approaches. In this review, we provide a summary of all commercially available rapid AST platforms for use in clinical microbiology laboratories. We describe the technologies utilized, performance characteristics, acceptable specimen types, types of resistance detected, turnaround times, limitations, and clinical outcomes driven by these rapid tests. We also discuss crucial factors to consider for the implementation of rapid AST technologies in a clinical laboratory and what the future of rapid AST holds.

## 1. Introduction

Antimicrobial resistance (AMR) is a major global health burden [1]. In 2019, 1.2 million deaths were attributed to infections caused by resistant organisms. After the COVID-19 pandemic, reports have revealed an increase in AMR of up to 50% [2]. According to the World Health Organization (WHO), certain antimicrobial agents, such as the third- and fourth-generation cephalosporins and carbapenems, are considered critically important as they are the last resort for the treatment of resistant infections. The rise in AMR has reduced the effectiveness of empirical antimicrobial treatments, prompting widespread adoption of antimicrobial susceptibility testing (AST) as a routine test in clinical laboratories. In the recent WHO’s Global Antimicrobial Resistance and Use Surveillance System (GLASS) report, bloodstream infections with resistant *Escherichia coli* and *Salmonella* species increased by 15% [3]. Among the bloodstream infections with *Klebsiella pneumoniae* and *Acinetobacter* species, resistance was detected in 50% of the cases, with 8% of *K. pneumoniae* isolates being carbapenem-resistant [3].

AST evaluates the bacteria’s response to an antimicrobial reagent in vitro and provides information about clinical therapeutic efficacy. In the clinical microbiology laboratory, the standard AST workflow begins after isolating and identifying the organism, which usually takes at least one day after the collection of specimens. After a pure isolate is obtained, different phenotypic (growth-based) AST approaches can be pursued, with results available 18–24 h later [4]. Consequently, at least two days are needed to obtain the susceptibility profile of an isolated bacteria from a clinical sample. Although these procedures are precise and reliable, they are time-consuming and labor-intensive. The gap in time before the susceptibility results are released may cause patients to be treated with suboptimal or ineffective therapy. It has been shown that there is a significant association between the delay in antibiotic administration with severe sepsis, septic shock, and increasing mortality [5]. Hence, rapid AST approaches are instrumental to improving patient care and clinical outcomes. In this review, we summarize the different types of rapid AST methods (<8 h) available for use in the clinical microbiology laboratory, technologies in development, and considerations for implementation of rapid AST in a clinical setting.

## 2. Conventional Approaches to Antimicrobial Susceptibility Testing

Bacterial organisms can be isolated in culture between 24 h to 1 week after the time of specimen collection. Conventional, phenotypic (growth-based) AST approaches are performed on pure, cultured isolates and can take up to 24 h before results are available. Gold standard phenotypic AST methods are broth and agar dilution, and other phenotypic AST methods include Kirby–Bauer (KB) disc diffusion and concentration gradient test strips like the E-tests [4,6].

The KB disc diffusion assay assesses bacterial susceptibility by measuring the diameter of the zone of inhibition around the antibiotic disk. Briefly, a standard inoculum of 0.5 McFarland (1.5 × 10^8^ CFU/mL) is used to inoculate a Muller–Hinton Agar (MHA) plate, in most cases. Antibiotic discs impregnated with a defined antibiotic concentration are placed on the MHA plate and then incubated for 18–24 h. The diameter of the zone of inhibition, also known as the clearing around the antibiotic disc, is then measured. The respective interpretations (e.g., S = susceptible, I = intermediate, or R = resistant) of the disk zone diameter is made according to clinical breakpoints established by the Clinical and Laboratory Standards Institute (CLSI) [7], United States Food and Drug Administration (FDA) [8], or the European Committee on Antimicrobial Susceptibility Testing (EUCAST) [9].

For agar and broth dilution assays, a standard bacterial inoculum is added to agar or broth containing antimicrobial agents that are serially diluted two-fold. Plates or broth are incubated overnight, and bacterial growth is assessed by visualizing bacterial growth or turbidity. The minimal inhibitory concentration (MIC), defined as the lowest concentration of an antimicrobial agent that inhibits bacterial growth in the broth or agar, can be determined. The Epsilometer test (E-test) consists of an antibiotic strip which contains a predefined gradient of an antimicrobial agent from one end to the other of the strip [10]. The strip is placed on an inoculated agar plate and is incubated overnight. The MIC is the point where an ellipse-shaped zone of inhibition intersects the E-test strip. The respective interpretations (e.g., S, I or R) based on the MIC are made accordingly to the CLSI, EUCAST, or FDA guidelines.

## 3. Rapid Phenotypic AST Approaches

In recent years, there has been an increase in the number of commercially available technologies providing rapid phenotypic AST. In Table 1, we list the methods that are currently approved by regulatory agencies and can be implemented for clinical use in either the United States (with Food and Drug Administration (FDA) clearance) and/or in European countries (with Conformite-Europeenne in vitro Diagnostic (CE-IVD) approval). Such technologies use sophisticated approaches such as microfluidics, morphokinetic cellular analysis, light scattering, fluorescence detection of viability or cellular damage, flow cytometry, and basic microscopy to measure or visualize bacteria growth under the presence of antibiotics.

While the methods all have a run time of <8 h, general differences among the platforms include the technology used, whether the technology is applicable to Gram-positive and/or Gram-negative organisms, and the specimen type. Most platforms are for positive blood cultures while only one (Selux Next-Generation Phenotyping (NGP) Test, (Boston, MA, USA)) can be performed on both positive blood cultures and cultured bacterial isolates. Most of the rapid phenotypic AST technologies, apart from the PhenoTest (Accelerate Diagnostics Inc., Tucson, AZ, USA) cannot identify the organism. The organism will need to be determined by another approach, such as a rapid molecular identification panel or direct identification on the matrix-assisted laser desorption/ionization time-of-flight mass spectrometry (MALDI-TOF). If definitive identification cannot be performed immediately, a presumptive observation based on the Gram stain can help decide whether the rapid phenotypic AST assay has the appropriate antibiotic panels.

To evaluate the performance of phenotypic AST, metrics such as essential agreement (EA), categorical agreement (CA), and errors such as very major errors (VMEs), major error (ME), and minor errors are evaluated. EA is defined as when the MIC result obtained with the AST system in evaluation (test method) is the same or within one doubling dilution step from the comparator (gold standard) method. CA is defined as when there are concordant interpretations (e.g., S, I, R) between the test and comparator method. VMEs are occurrences when the interpretations from the test method are susceptible but the comparator method is resistant. MEs are occurrences when the interpretations from the test method is resistant but the comparator method is susceptible. Minor errors are occurrences when one method interprets the isolate as I, but the other method interprets the isolate as S or R. General guidance for acceptable accuracy consists of EA and CA ≥90%, VME of <3%, and ME of <3%, while the percentage of minor errors can be determined by the laboratory director [11]. According to Cumitech, the combined performance for major and minor errors <7% is allowed [11]. A CA of <90% can be acceptable if most errors are minor errors and have essential agreement.

The PhenoTest BC (Accelerate Diagnostics Inc., Tucson, AZ, USA) was one of the first platforms to acquire FDA clearance and CE-IVD approval. The PhenoTest has several advantages. First, the PhenoTest can perform both rapid identification of the organism in positive blood cultures as well as rapid AST. Identification is carried out by fluorescence in situ hybridization within 1.5 h. AST can be achieved in 7 h and is performed by a proprietary ‘morphokinetic’ approach involving time-lapse microscopy imaging of the bacteria. Comparing the morphological and kinetic changes in the bacteria under antibiotic treatment conditions to the untreated growth control allows for the determination of the MICs. The PhenoTest can identify Gram-positive bacteria, Gram-negative bacteria, and yeasts (*Candida albicans* and *Candida glabrata*) and perform AST for Gram-positive and Gram-negative bacteria. Given that the PhenoTest has been on the market for >5 years, the platform has been widely studied. The PhenoTest’s accuracy in pathogen identification ranges from 87 to 100% [12,13]. The highest performance was observed in Enterobacterales, followed by *Staphylococcus* species and *Enterococcus* species. Accurate identification of organisms in polymicrobial cultures is variable. Some studies have found that 100% (10/10) polymicrobial cultures were correctly identified, while other studies reveal accuracy as low as 12.5% (3/24) [14,15]. Overall, the CA and EA were both >91%, with better performance in the Gram-positive bacteria than the Gram-negative bacteria [12,14,15,16,17,18]. For the Gram-positive bacteria, the CA and EA were 92–99% and 82–97%, respectively, whereas for the Gram-negative bacteria, the CA and EA were from 90–99% and 91–95%, respectively [12,18].

The LifeScale (Affinity Biosensors, Santa Barbara, CA) test, with a run time of <5 h, utilizes a technology that does not rely on the detection of bacterial growth or metabolism. The instrument uses microfluidic sensors to create a ‘Population Profile’ that is equivalent to the mass of the bacteria in the sample [19]. The data are then fed into an ‘AI Predictor’, which can determine the MIC of the antibiotic for the tested organism. Evaluating 665 drug–bug combinations, a comparison study revealed an EA and CA of 95.3% and 93.1%, respectively [20].

The ASTar (Q-linea, Uppsala, Sweden), with a run time of <7 h, utilized high-speed time-lapse microscopy at different intervals to image bacterial growth in the cultured chambers prefilled with different antibiotics. The images are used to calculate bacterial biomass in order to determine the MIC. The ASTar received CE-IVD approval but recently received U.S. FDA 510(k) clearance as of April 2024. Across three evaluation studies, the EA and CA were 90–98% and 95–97%, respectively. The rate of major errors and very major errors were 0.9–2.5% and 2.4–3.3%, respectively [21,22,23]. Common errors across the studies were seen in amoxicillin/clavulanic acid and piperacillin/tazobactam.

The VITEK REVEAL (bioMerieux, Mountain View, CA, USA), with a run time of <7 h, utilized sensors to detect changes in the volatile organic compounds that are emitted from bacteria during growth. The advantages of this platform are that the laboratory can see real-time monitoring of MICs and has the option to test up to 177 drug/bug combinations. The VITEK REVEAL received CE-IVD approval but recently received U.S. FDA 510(k) clearance as of June 2024. Using randomly selected prospective and contrived positive blood cultures with highly resistant isolates from the CDC/FDA Antibiotic Resistance Isolate Bank, the VITEK REVEAL had an EA and CA of 98.0% and 96.3%, respectively, and a very major error of 1.3% [24].

The Selux NGP Assay (SeluxDX, Boston, MA, USA) requires several instruments for testing depending on whether the specimen is a blood culture or bacterial isolate. For AST directly from positive blood cultures, the blood culture is placed in the Selux Separator, which isolates the bacteria from the blood and prepares an AST-ready McFarland equivalent to be placed into the Inoculator instrument, where the sample will be dispensed into their appropriate antibiotic panels. For testing from direct colonies, users need to prepare a McFarland inoculum before bringing the sample prep carrier to the Inoculator instrument. After bacteria inoculation, the plates are then placed into the Analyzer instrument where AST is performed. After sufficient growth is achieved in the wells, viability and surface area assays are performed. Viability assays utilize fluorescent markers to determine the metabolic activity of the bacterial population. Surface area assays are performed to determine any morphological changes (‘shape shifters’) on the bacterial surface in response to antibiotics [25]. The Selux NGP Assay can be modified into a very high-throughput workflow since the 384-well plate contains much room to accommodate other antibiotics or adjust for updated breakpoints if needed. The panel plate also includes multiple control wells to ensure growth and accuracy. As of the writing of this review, the panels differ depending on the tested specimen. There are 22 drug–bug combinations for Gram-negative isolates, 13 combinations for Gram-positive isolates, and 17 combinations for positive blood culture bottles with Gram-negative organisms. The Selux NGP Assay (SeluxDX, Boston, MA, USA) received CE-IVD approval but recently received U.S. FDA 510(k) clearance as of February 2024. Overall, great performance has been reported, with EA and CA >95% and major errors and very major errors at <1% [25,26].

The QuickMIC (Gradientech, Uppsala, Sweden) system is a microfluidic system that allows for the incubation of the bacteria in a gel [27]. Positive blood culture specimens are mixed in a bacteria–agarose mix and placed into a chip. The bacteria are exposed to a linear, diffusion-limited, gradient of antibiotics. Microscopy is used to monitor the growth rate of the microcolonies and the zones of inhibition over time. The benefit of this system is that rather than testing antibiotic concentrations in doubling dilutions like other typical phenotypic tests, QuickMIC can provide precise MIC values. Better optimized pharmacokinetics/pharmacodynamics dosing can be achieved in the patient with precise values of MIC reported. Currently, with only the CE-IVD status, the QuickMIC System recently received the FDA Breakthrough Device Designation in July 2023. A study with both spiked and prospective clinical blood cultures revealed variable performance depending on the antibiotic and/or bacteria tested. In spiked specimens with reference isolates, the EA between QuickMIC and broth microdilution is 83.4%, with a range from 70 to 91%. The CA was 87.4% with a range from 57 to 99%. In true clinical specimens, the EA ranged from 45 to 100% and the CA ranged from 78 to 100% [28]. Low EA (45–87%) and CA (57–78%) was seen for tigecycline. Additionally, the non-fermenters performed more poorly than the fermenters. Non-fermenters had an EA and CA of 68–79% and 82–86%, respectively, compared to the fermenters with an EA and CA of 84–91% and 87–95%, respectively.

Alfred 60/AST (Alifax, Padova, Italy) platform monitors growth of bacteria by using light scattering technology via a photodetector directing light at different angles (30° and 90°). This instrument can provide information regarding the growth rate of the isolate by using the growth control to develop a reference curve [29]. A caveat of this platform is that MIC determination is not available; only CA can be evaluated compared to traditional approaches. However, unlike other rapid phenotypic AST platforms, the Alfred 60/AST can test both Gram-positive and Gram-negative organisms. The overall CA is >94% but, when broken down by the organism, the CA of Gram-negative organisms range from 91 to 95% and the CA for Gram-positive organisms range from 88 to 95% [30,31,32,33]. Discordances were seen in piperacillin–tazobactam across multiple studies. The time to result varies anywhere between 4 and 7 h which is dependent on the drug–bug combination.

dRAST (QuantaMatrix, Seoul, South Korea) uses microscopic imaging to determine the MIC of the antibiotics. Their patented technology consists of immobilizing the bacteria in a gel matrix followed by hourly imaging. Bacterial growth is determined by the number of colonies grown in the agar and the changes in colony size over time. CA and EA of 91–92% and >95%, respectively, with minor errors of 4.8–6.6%, major errors of 2.7–3.5%, and very major errors of 1.45–2% were reported [34,35,36,37]. Antibiotics with higher rates of errors include gentamicin, piperacillin–tazobactam, and the combination of cefoxitin/oxacillin for *Staphylococcus*. One advantage of the Gram-negative panel is the inclusion of a test for Extended Spectrum Beta-Lactamase (ESBL) producers. In a study with 52 isolates, a false positive rate for ESBL-detection of 5.8% was reported; this included a *K. oxytoca* K1 strain, a *K. pneumoniae* hyperproducing SHV-1 and a wild type *P. mirabilis* [37]. However, others have reported very major error rates of 3.3–13.3% [35,36].

FASTinov (Porto, Portugal) is another non-growth-based approach for rapid phenotypic AST. FASTinov utilizes flow cytometry and fluorescent probes to identify bacteria cellular changes such as cell lesions or metabolic alterations in the presence of antibiotics. Different flow cytometry instrumentation (CytoFlex model B3-R0-V3 (Beckman Coulter, Brea, CA, USA) and the DxFlex (Beckman Coulter, USA)), detecting different wavelengths, have been shown to have great performance using the FASTinov kits. Of note, both flow cytometers are equipped with one blue laser (488 nm; output, 50 mW; beam spot size, 5 by 80 μm) [38]. CA of >96% was achieved for both Gram-positive and Gram-negative organisms. Detection of ESBL producers by the FASTinov had a sensitivity and specificity of >96% and 100%, respectively [38,39]. The FASTinov Gram-negative panel also has a screening for the AmpC plasmid, with both sensitivity and specificity of 100%. Carbapenemase screening also showed sensitivity and specificity of >92%. An advantage of the FASTinov panels is the inclusion of colistin for the Gram-negative organisms, which has a CA of >99% [40,41]. Although FASTinov is used for AST, there have been attempts to modify the FASTinov workflow to allow for rapid identification of the organism. The bacterial suspension from the FASTinov sample preparation can be repurposed and be tested directly on the MALDI-TOF for rapid identification of the organism directly from positive blood culture bottles [42]; the agreement in the identification of Gram-positive and Gram-negative organisms was >95%. However, this is considered off-label use and hence would require a laboratory to perform a full validation.

For laboratories who do not have the resources to invest in new technologies, there has been an attempt to develop and standardize a modified version of the KB disk diffusion test as a rapid phenotypic AST approach directly from positive blood cultures [43,44]. The direct blood culture disk diffusion test is a rapid phenotypic test that delivers results directly from positive blood culture broths after 8 to 10 h or 16 to 18 h (depending on the antibiotics) of incubation. Unlike conventional AST, it does not require the isolation of pure colonies thus providing results 22 h earlier. This test is performed concurrently with a rapid identification test, such as a rapid molecular test or MALDI-TOF MS [45]. Thus far, standardized procedures are only available for positive blood culture broth with Gram-negative bacilli, specifically Enterobacterales, *P. aeruginosa*, and *Acinetobacter* species. This procedure is to be performed within 8 h after the blood culture bottle flags positive. Briefly, a total of 12 drops of blood (from a 20-gauge venting needle) are spread across the surface of the MHA. The appropriate antibiotic discs (as determined by the organism) are then dispensed onto the surface of the inoculated plate and incubated for either 8–10 h or 16–18 h. For full procedures and specifics of the drug–bug combinations, please refer to CLSI M100 Table 3F-1. Test for Performing Disk Diffusion Directly From Positive Blood Culture Broth [7]. This highly accurate method can be used on non-fastidious pathogenic bacteria and provide susceptibility results one day earlier [46]. It is low cost and can be easily implemented in laboratories that already use KB disk diffusion in-house. To further decrease turnaround time, total laboratory automation instrumentation may assist with the setup and reading of results.

## 4. Rapid Genotypic AST Platforms

Genotypic AST methods use nucleic acid amplification technologies, such as polymerase chain reaction (PCR) and DNA microarray, to detect specific AMR genes. These techniques are generally employed for direct, rapid, sensitive, and specific detection of AMR genes [47,48]. They can also minimize the need for laborious and time-consuming bacterial cultures and reduce the chances of contamination.

Surveying 96 laboratories in the United States, 90/96 (94%) of the laboratories indicated that they used a rapid molecular platform [49]. There are many options of rapid genotypic tests commercially available. Technologies utilized include PCR, fluorescence in situ hybridization, or microarrays. Some assays are for diagnostic purposes while others are for infection control purposes. Specimen types vary from positive blood cultures, respiratory specimens, swabs, stool, and cultured isolates. Even though there are multiple tests detecting resistance for the same classes of antibiotics, the actual genetic target used can also vary among different assays and manufacturers. Assays may be more targeted for a specific pathogen (e.g., singleplex panels) or can be used more broadly in the context of specific syndromes (e.g., multiplex, syndromic panels). Run time of these tests range from 45 min to 5 h. Table 2, Table 3, Table 4 and Table S1 describe the different commercially available rapid genotypic AST tests.

### 4.1. Genotypic Approaches with Limited On-Panel Targets

While many laboratories utilize multiplex panels (>14 targets) for positive blood cultures, there are also commercially available genotypic tests for positive blood cultures detecting fewer targets, such as only *S. aureus* and/or MRSA (Table 2). The AMR targets do vary depending on the manufacturer and can consist of *mecA/C*, *SCC mec/attB*, *SCC mec/orfX*, or MREJ. The caveat of these tests is that in the presence of a polymicrobial culture consisting of both *S. aureus* and a coagulase-negative *Staphylococcus*, it is difficult to discern whether methicillin-resistance is attributed to the *S. aureus* or coagulase-negative *Staphylococcus*. As such, ‘detection of *S. aureus’* with ‘*mecA* gene not detected’ should be interpreted as ‘MRSA indeterminate’, given that there may be other *mec* genes (e.g., *mecC*) conferring resistance to methicillin not detected by the assay [50,51].

Singleplex testing can also be performed on skin/soft tissue (from surgical site, diabetic foot wounds, abscesses, and cellulitis), nasal, peri-anal, and rectal swabs (Table 2). It is important to note that not all of these are cleared for utility in diagnostic purposes but used instead for infection control, isolation, and surveillance purposes. The advantage of these tests is the identification and detection of resistance genes without requiring bacterial growth to be performed within several hours. This allows for the rapid isolation of colonized patients and the execution of infection control protocols to prevent nosocomial infections at the start of admission. For methicillin resistance, these assays detect the *mecA/C* gene and/or the insertion of *SCCmec* into *attB* or *orfX.* For vancomycin resistance, these assays detect the *vanA/B* gene. For carbapenemase production, these assays detect the *blaKPC*, *blaIMP*, *blaNDM*, *blaVIM*, or *blaOXA-48* genes.

There are two assays that are commercially available for the detection of AMR genes from cultured bacterial isolates (Table 2). The Revogene Carba C (formerly GenePOC Carba assay) (Meridian Bioscience, Cincinnati, OH, USA) can detect the common carbapenemase genes from cultured isolates of Enterobacterales, *Pseudomonas aeruginosa*, and *Acinetobacter baumanii* within 70 min. Meanwhile, the Acuitas AMR Gene Panel (OpGen, Rockville, MD, USA) is a comprehensive PCR test that can detect resistance to nine classes of antibiotics from cultured isolates of Enterobacterales, *Pseudomonas aeruginosa*, and *Enterococcus faecalis* (Table 2 and Table 3). The panel includes 28 AMR markers indicative of resistance to the following classes: aminoglycosides (*aac*, *aad*, *ant*, *aph*, *armA*, *RMT*), carbapenems (*blaCMY*, *DHA*, *blaIMP*, *blaKPC*, *blaNDM*, *blaOXA-48*, *blaPER*, *blaVIM*), cephalosporins (*blaCMY*, *blaCTX-M-1*, *blaCTX-M-2*, *blaCTX-M-9*, *DHA*, *blaOXA-9*, *VEB*), fluoroquinolones (*gyrA*), vancomyin (*vanA*), penicillins (*blaOXA-1*, *blaSHV*, *blaTEM*), colistin (*mcr-1*), sulfonamides (*sul1*, *sul2*), and trimethoprim (*DFR*).

A verification/validation of molecular methods typically include metrics such as sensitivity (or positive percent agreement [PPA]) and specificity (or negative percent agreement [NPA]) [52,53]. A true positive result is when the genotypic test detects the presence of an AMR gene in an isolate that is either phenotypically resistant to the antibiotic or, in some instances, shown to be resistant to the drug by other molecular methods (e.g., whole genome sequencing). A true negative result is when the genotypic test does not detect the AMR gene in an isolate that is phenotypically susceptible or is proven to not harbor the AMR gene by other methods (e.g., whole genome sequencing). Sensitivities and specificities ≥90% are typically acceptable.

The Acuitas AMR gene panel is the first FDA-cleared panel to include such broad AMR markers. While whole-genome sequencing methods can also offer a comprehensive profile of AMR genes, the Acuitas AMR gene panel is not as cumbersome and does not require as much technical expertise. Briefly, DNA from the bacterial isolates is extracted followed by real-time PCR. Data from the PCR runs are analyzed using the Acuitas AMR gene analysis software, which generates a report with the resistance genes marked as “detected” or “not detected”. A multicenter study evaluating 1224 isolates reported that the positive percent agreement (PPA) and negative percent agreement (NPA) ranged from 94 to 100% and from 96 to 100%, respectively [54]. The overall Acuitas AMR gene panel workflow from sample processing to generation of report is 2.5 h.

The Xpert MTB/RIF assay (Cepheid, Inc., Sunnyvale, CA, USA) was endorsed by the WHO in 2010 as a reliable diagnostic tool to detect *Mycobacterium tuberculosis* and identify rifampin resistance by PCR amplification of the rifampin resistance-determining region (RRDR) of the *rpoB* gene and probing for mutations associated with rifampin resistance. Compared to standard cultures, which can take up to 6 weeks for *M. tuberculosis* to grow and another 3 weeks minimum for AST, the Xpert MTB/RIF assay revolutionized the diagnosis of *M. tuberculosis* and subsequently played a significant role in treatment and infection control of tuberculosis. The assay has been adopted in >130 countries, including low resource settings [55,56,57]. Performance of the assay varies depending on smear positivity, patient status (e.g., with paucibacillary disease or HIV disease), and the prevalence of tuberculosis in each geographic location [58,59,60]. Cepheid, Inc. developed the Xpert MTB/RIF Ultra assay to improve sensitivity in detection of *M. tuberculosis* and rifampin resistance. Despite these new efforts, multicenter studies reveal that the performance for detection of rifampin resistance was comparable to the older version; the original Xpert MTB/RIF assay showed sensitivity and specificity of 80–98% and 94–100%, respectively, compared to the Xpert MTB/RIF Ultra with sensitivity and specificity of 80–98% and 92–99%, respectively [61,62]. Although the manufacturer only validated the test for sputum specimens, individual laboratories have made efforts to utilize this assay on extrapulmonary specimens such as cerebrospinal fluid, bone and joint, lymph node specimens, bodily fluids, as well as directly from MGIT broth cultures that are positive for acid-fast bacilli [60,63].

### 4.2. Genotypic Approaches for Syndromic, Multiplex Testing

There are several molecular platforms available for detection of AMR genes from positive blood culture bottles (Table 4). Technology varies from PCR amplification to DNA hybridization. Some assays such as the blood culture identification panels (BCID) from DiaSorin (Saluggia, Italy) and GenMark Diagnostics (Carlsbad, CA, USA) have individual Gram-positive versus Gram-negative panels. There are many studies evaluating the performance of these BCID panels, with all suggesting that sensitivity and specificity for most targets are >95% [64,65]. In all platforms, the common AMR genes on the panels are those that confer resistance to methicillin, vancomycin, production of ESBLs, and carbapenem resistance, but some additional genes are unique to certain panels. The BioFire FilmArray BCID2 (bioMerieux, Marcy-l’Étoile, France) panel includes MREJ as a molecular target to specifically link the *mecA/C* gene to *Staphylococcus aureus* to identify methicillin-resistant *Staphylococcus aureus* and the *mcr-1* gene for colistin resistance.

Both the BioFire FilmArray Pneumonia Panel (bioMerieux, Marcy-l’Étoile, France) and the Unyvero LRT BAL Application (Opgen, Rockville, MD, USA) are multiplex molecular panels used on lower respiratory tract specimens such as sputum and BAL. The PPA for resistance detection was 80–100% [66,67]. Aside from the routine AMR genes for methicillin resistance, carbapenem resistance, and ESBL producers, the Unyvero LRT BAL Application also includes *blaTEM* for ESBL detection and *blaOXA-23*, *blaOXA-24*, and *blaOXA-58* carbapenamases. Of note, the targets for detection of MRSA differ between the two panels as the BioFire FilmArray Pneumonia Panel includes the MREJ linker. Pneumonia panels are not as widely implemented compared to BCID panels because organisms detected from non-sterile specimen types (e.g., sputum) may be indicative of colonized bacteria which could lead to unnecessary usage of antibiotics [68]. Additionally, detection of an AMR gene is not linked to the organisms detected.

Currently there is only one commercially available multiplex panel with regulatory clearance for joint infections; the BioFire FilmArray Joint Infection Panel (bioMerieux, Marcy-l’Étoile, France) can detect AMR genes for methicillin, vancomycin, and carbapenem resistance directly from synovial fluid [69].

## 5. Phenotypic versus Genotypic Approaches

**Figure 1:**
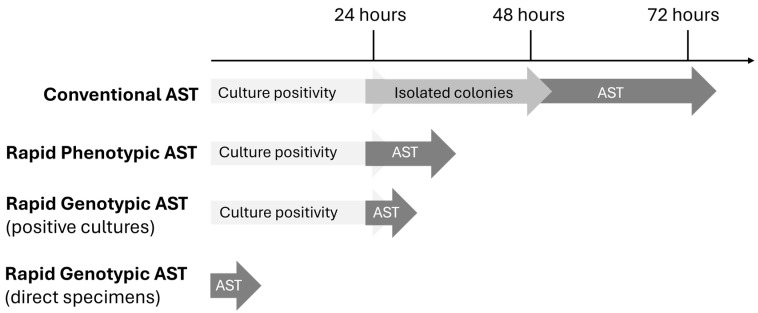
Different antimicrobial susceptibility testing approaches and their potential time-to-result. Abbreviations: AST, antimicrobial susceptibility testing.

Genotypic approaches are not meant to replace phenotypic testing but are used to supplement one another due to their differences (Table 5). Phenotypic approaches determine the susceptibility of the antibiotic by evaluating growth and/or bacterial metabolic changes in the presence of the antibiotic. In most cases, phenotypic AST approaches are performed after the organism is isolated in pure culture which could take up to a few days. They have the advantage of demonstrating both qualitative and quantitative antimicrobial susceptibility to antimicrobial agents by interpretations (e.g., S, I, R) and MIC values, which are important for therapeutic decisions. Phenotypic approaches can most often be easily adapted for the testing of newer antimicrobials or when genetic mechanisms of resistance are unclear, especially for Gram-negative organisms. In contrast, genotypic AST methods depend on the established knowledge of AMR genes or mutations. These tests can be performed directly from specimens, greatly reducing the time to diagnosis (Figure 1). Some of the molecular assays can even identify the organisms in addition to performing AST. Molecular panels that offer both identification and AMR gene detection are typically syndromic panels (with >14 targets), which are more costly and subject to reimbursement concerns. A significant limitation of genotypic AMR detection is its reliance on subsets of molecular targets, meaning the results depend on the available list of targets and the known mechanisms of resistance. Despite a positive genotypic result, concomitant cultures are necessary for further epidemiological typing or susceptibility testing of off-panel antibiotics. Genotypic methods detect the presence or absence of a gene but do not determine if the gene is functional, which can lead to overcalling resistance. Genotypic AST tests predict resistance, not susceptibility, so a lack of genes detected may not confidently suggest that the isolate is susceptible. Additionally, molecular methods are less accurate in detecting polymicrobial infections and have difficulty in discerning which organism(s) harbor the AMR gene(s) detected [70].

Results from genotypic AST approaches very often provide AMR information prior to phenotypic AST (Figure 1). In a handful of cases, discrepancies can arise between the genotypic and phenotypic susceptibility results. Discrepant results can occur when an AMR gene is detected in an isolate that is phenotypically susceptible to the predicted agents or when an AMR gene is not detected, but the isolate is found to be phenotypically resistant to the predicted agents. It is imperative for clinical laboratories with both genotypic and phenotypic platforms to implement protocols addressing potential discordant results to prevent adverse events, reduce inappropriate therapy, and improve antibiotic stewardship [71].

Discrepancies can occur due to biological, technical, or clerical causes. An important consideration is that genotypic methods detect limited molecular targets, and resistance can be caused by other mechanisms of resistance. This principle especially applies to Gram-negative organisms with complex resistance mechanisms, ranging from specific mechanisms such as carbapenemase production, ESBL producers, to broader mechanisms such as porin mutations and efflux pumps. For example, an Enterobacterales may not have the *blaKPC or blaCTX-M* gene detected but may still show phenotypic resistance [72]. In instances of polymicrobial infections, genotypic methods may not be able to precisely attribute a resistance mechanism to a specific organism in such cases. Technical causes such as nucleic acid contamination and clerical errors performed during reporting are also worth considering during the troubleshooting process. It is now a College of American Pathologists (CAP) requirement (CAP checklist item MIC.21855) to link AMR genes and phenotypic susceptibility results to a specific organism in the final patient report. The Clinical and Laboratory Standards Institute (CLSI) have published guidelines (appendix H, M100 standard Use of Molecular Assays for Resistance Detection) for resolving discrepancies [7,71]. If a discrepancy is unable to be resolved, it is recommended to report the isolate as resistant and consider further discussion with the Infectious Diseases or antimicrobial stewardship team.

## 6. Clinical Impact

Rapid AST has the potential to improve clinical outcomes compared to standard of care methods by providing clinical teams with the knowledge to optimize antimicrobial therapy [73,74]. Studies have shown that a variety of rapid AST approaches, both phenotypic and genotypic, can lead to decreased time to antimicrobial changes, lower mortality, shorter duration of stay in the hospital and intensive care unit, and reduced costs. However, the findings are not always consistent among the studies. The limitations of these studies are that they are retrospective, observational, and single-center studies that evaluate the clinical impact pre- versus post-implementation of rapid AST approaches without the appropriate control groups.

There are only a handful of randomized controlled trials that compare the clinical impact of rapid blood culture AST methods and standard of care approaches. Banerjee et al. report in their single-center, randomized, controlled trial where patients (N = 617) with positive blood cultures were randomized into three separate groups—conventional testing, receiving the BCID test, or receiving the BCID test plus antimicrobial stewardship review. Patients with BCID testing had a faster time to antibiotic escalation, less treatment of blood contaminants, and less use of broad-spectrum antibiotics. Moreover, patients with BCID and antimicrobial stewardship review, which consisted of 24/7 audit and feedback by an infectious disease pharmacist or physician, had more antibiotic de-escalation than those who received BCID only. No differences were seen in mortality, length of hospital stay, adverse events, or cost of care [75]. Of note, the rapid BCID test had a greater impact on the management of Gram-positive infections than Gram-negative infections. In another multi-center, randomized control trial with two arms evaluating the impact of the PhenoTest (Accelerate Diagnostics, Tuscon, AZ, USA) compared with standard of care approaches (N = 448) revealed that the study arm with the PhenoTest had decreased turnaround time and had a faster time to antimicrobial optimization but no significant changes to mortality, length of stay, adverse events, or cost [74].

To date, there are limited randomized controlled trials evaluating the impact of multiplex lower respiratory pneumonia PCR panel, especially in terms of the utility of its AMR markers. For one study, active antimicrobial stewardship recommendations resulted in 45% of inappropriate antibiotic therapy but no differences in overall duration on antibiotics, length of stay and discharge, or ICU admission rate [76]. Another single-center, retrospective study revealed that discontinuation of MRSA and anti-pseudomonal therapy were faster upon implementation of the pneumonia panel (although insignificant) and the results were driven by involvement with antimicrobial stewardship [77]. More studies are needed to determine the clinical impact of the pneumonia panel with AMR markers, but it is safe to say, as with the findings from blood culture identification panels, collaboration with antimicrobial stewardship most likely increases positive clinical impact.

Meanwhile, another clinical application of rapid AST, especially genotypic testing, is for infection control and prevention purposes. Rapid genotypic testing allows for prompt execution of isolation procedures to prevent nosocomial infections. Rapid tests allow for surveillance and prompt identification of infected patients or potential colonizers. High-risk patients, such as those who are immunocompromised, undergoing dialysis, or those with chronic underlying comorbidities, have frequent hospitalizations and/or are in contact with infected patients, may be subject to screening. Also, the published guidelines recommend a nasal screening for MRSA to decide the treatment plans for non-severe pneumonia, pre-operative decolonization, or perioperative antibiotic prophylaxis [78,79]. A benefit of these genotypic tests is that they are easy to use, and manufacturers have validated their tests for non-invasive specimen types appropriate for screening purposes, such as nasal swabs or rectal swabs.

For example, a study comparing the impact of two different control measures (real-time PCR for vancomycin resistant *Enterococcus* versus chromogenic selective medium as standard of care) was performed. The turnaround time was 70.5 h for the specimens tested using the chromogenic media compared to the turnaround time of 4.6 h for RT-PCR. Additionally, >40 cases would have been missed in the standard of care group. The estimated cost of RT-PCR was EUR 870, whereas the estimated cost due for testing and interruption of admissions using standard of care approaches was EUR 14,302 [80]. In another prospective, single-center study, the utility of the rapid PCR testing in controlling nosocomial spread of carbapenem-resistant organisms in a hospital setting was evaluated in the ICU. Rectal swabs were collected from all the patients from the beginning of their stay in the ICU ward and once weekly until discharge. Implementation of rapid surveillance testing led to a reduction in both colonization by 23% and infection by 32.9% [81].

## 7. Considerations for Implementation of Rapid AST Technologies in the Clinical Laboratory

Clinical laboratories have much to consider when it comes to implementing a rapid AST system. The diverse types of technologies, their range of targets, performance characteristics, time to result, and clinical impact have been described above, but there are many other factors worth mentioning. The new assay to be implemented must be adaptable for the current clinical laboratory workflow and can enhance the care of each institution’s unique patient population (Table 6).

As the rates of antimicrobial resistance continue to rise, it is becoming more important to be able to test for a breadth of different drug–bug combinations to ensure that the clinical teams have options to treat their patients. Platforms such as the Selux NGP test (SeluxDX, Boston, MA) which allow for customization of the panel and inclusion of new antibiotics offer a potential advantage. Additionally, each laboratory is required to adhere to different guidelines for interpretative criteria of AST. For laboratories in the United States, most follow CLSI or FDA breakpoints, whereas those in European countries follow EUCAST breakpoints. For laboratories who are CAP-accredited, there is now a CAP checklist item MIC.11385: Current Antimicrobial Susceptibility Test Interpretation Breakpoints that states ‘Effective January 1, 2024, the laboratory uses current breakpoints for interpretation of antimicrobial minimum inhibitory concentration (MIC) and disk diffusion test results.’ New breakpoints (by the FDA, CLSI, or EUCAST) are to be implemented within three years after publication. Manufacturers that produce technology with the goal of serving a diverse international market need to ensure that their instrumentation meets the regulatory standards of the respective regulatory agencies. To require the laboratories to validate or implement off-line alternatives will be a challenge as it requires laboratory expertise, potential changes in laboratory workflow, as well as regulatory hurdles [82]. Additionally, a known limitation for molecular panels is the limited list of on-panel targets, usually only accounting for common resistance mechanisms. Multiplex molecular panels have added complexity which increases the risk of technical issues such as primer mispairing, nonspecific amplification, and primer dimer formation, which can compromise the sensitivity of the test [83].

Laboratory requirements for space, supplies, and consumables can impact purchasing decisions. Physical space restraints are an area of concern. For example, the Selux NGP Test requires at least two instruments, an Inoculator and Analyzer. Each instrument is at least 62 cm × 74 cm × 90 cm and needs to be placed on the ground, taking up space in the laboratory. Additionally, depending on the test volume in each laboratory, the flexibility in the modularity of the instruments to allow for laboratories to increase testing throughput is especially important to ensure continuity of workflow. As we continue to face a dwindling workforce in laboratory medicine, it would be helpful to implement assays that require low expertise, low maintenance, and can be easily integrated into the current workflow. For example, the VITEK REVEAL is part of the bioMerieux’s portfolio of products; their phenotypic rapid AST solution can be integrated with their other sepsis workflow products such as their blood culturing system, molecular rapid identification panel, routine antimicrobial susceptibility platforms, and data analysis. This allows for their clinical laboratory clients to have streamlined, continuous workflow for patient testing, but is also beneficial for customer support and maintenance from a laboratory management perspective.

It is important for laboratories to perform their own validation to determine the real-world experience and the best approach to incorporate the new technologies into their current workflow. For example, published studies have demonstrated that internal validation and ‘real-world’ evaluation of the PhenoTest (Accelerate Diagnostics Inc., Tucson, AZ, USA) compared to the standard of care AST platform influenced how the PhenoTest would be incorporated into laboratory workflow. Truong et al. showed that most of the minor errors were overcalling resistance; in 64.5% of the cases, the PhenoTest called intermediate when the reference was susceptible, and in 25.8% of the cases, the PhenoTest called resistant when the reference was intermediate. Due to the overcalls of resistance, their laboratory implemented an algorithm where intermediate or resistant results were not released and were to be confirmed by the reference method [84]. Similarly, as described in Patel et al., they found that for *P. aeruginosa*, VMEs of >40% were seen for cefepime and ciprofloxacin and MEs of >19% were seen for piperacillin–tazobactam and ceftazidime. Since their medical center could not evaluate the accuracy of less encountered organisms such as *Acinetobacter*, they decided to only release the results for selected antibiotics for Enterobacterales only [85].

The published turnaround time may reflect the time to result once the specimen is on the instrument as opposed to the actual turnaround time for a particular institution, given the differences in laboratory workflows and operating hours. In European countries where some of these technologies are developed and hence where original published studies are performed, it is important to note that their clinical laboratories may not be 24/7 laboratories, and thus published turnaround times may not be appropriate for all laboratories [30]. One must also consider the staffing schedule and the hours of other stakeholders, such as the antimicrobial stewardship teams, unless the hospital utilizes automated notifications. Integrating these new assays into clinical practice may require changes in the laboratory workflow, comprehensive training for laboratory personnel, and implementation of decision-making processes (e.g., electronic, automated communication to antimicrobial stewardship teams and providers). New workflows and reporting structures may cause resistance from laboratory staffing and healthcare providers accustomed to established diagnostic methods.

The reporting of phenotypic susceptibility results has been relatively straightforward and is routine for many clinical laboratories. Given the trust and comfort of this standard method for many decades, clinical teams using this information rarely have questions. However, it must be of noted that aside from the common interpretations of S (susceptible), I (intermediate), and R (resistance), there is also S-DD (susceptible-dose dependent), which is dependent on the antibiotic and specific dosing recommendations [7]. Reports with S-DD could benefit from an explanation or footnote for the clinical teams who are not used to this designation. Additionally, every year, there are updated breakpoints and interpretations by CLSI, EUCAST, or FDA. However, not every laboratory has breakpoints updated. Using updated breakpoints allows for more appropriate optimization of therapy due to the latest information regarding effectiveness of the selected antibiotics or changing epidemiology of resistance. Among the CAP-accredited laboratories, 70% of U.S. laboratories and 45% of laboratories outside the U.S. use obsolete clinical breakpoints to interpret phenotypic AST results [86]. In laboratories using obsolete breakpoints, over 50% had no plans to update to current breakpoints, citing manufacturer-related issues and the lack of resources and expertise to perform the analytical validation studies. It is paramount that there is communication between the laboratories performing AST and the clinical teams using the information to understand the limitations of the methodologies and how the results are reported.

Incomplete understanding or lack of knowledge regarding the antimicrobial resistance mechanisms and genes may lead to missed opportunities for antimicrobial optimization when rapid genotypic AST panels are used. In a survey consisting of 156 physicians to assess physicians’ interpretation of molecular results from a rapid BCID panel, the correct response rates for result interpretation questions ranged from 52 to 86% [87]. The study suggested that the adoption of new technology also required improvements in reporting. Although laboratories are required to report what manufacturers have on their report, which is typically ‘detected’ or ‘not detected’, laboratories should include a comment to help clinical teams reading the report to interpret the results [49,70]. The final report should link the AMR gene to the organism with interpretative guidance. For example, ‘presumptive methicillin-resistant *S. aureus’* should be in the report if the *Staphylococcus aureus* and the *mecA* targets were detected as opposed to merely reporting ‘detected’ for two targets. In some instances, laboratories may also include a note to refer to consultation with infectious diseases or antimicrobial stewardship. Some laboratories may also include a note for treatment recommendations; for example, when *blaCTX-M* is not detected, a treatment recommendation can be ‘ceftriaxone is recommended for initial therapy pending susceptibility results.’ Any templated comments, or routine treatment recommendations should be made collectively with agreement among all stakeholders including the infectious diseases team, pharmacy, and antimicrobial stewardship teams. For this reason, there may be hesitation in bringing rapid AST approaches into point of care settings since positive clinical outcomes are usually associated with an active, on-site stewardship team.

Lastly, implementing new assays involves significant economic challenges. The initial costs are substantial, including the purchase of advanced equipment and specialized reagents. There is a continuous need to justify the financial and clinical benefits within the competitive environment of hospital resource allocation. Navigating the complex reimbursement systems for both inpatient and outpatient settings adds another layer of difficulty, making it challenging to secure financial compensation. The cost of other platforms is not readily available for a thorough review in this manuscript, but it is important to take factors such as test volume commitment and reagent rental plans versus capital purchase into consideration as these can affect the price. Additionally, manufacturers of newly approved assays may offer Early Adoption Program to help ease the clients into purchasing the equipment. Although the rapid turnaround time for the AST methods described here have great potential in a point of care setting, cost will be a big burden, especially in low resource settings. For example, the GeneXpert diagnostic platform (Cepheid, Inc.), which can perform several rapid genotypic AST tests (see Table 2 and Table 3), reported at least a total of $250 million in public investment to develop its original technology. Given that the Xpert MTB/RIF assay test has high potential in low resource settings, the Foundation for Innovative New Diagnostics (FIND) entered into a Cooperative Research and Development Agreement with Cepheid Inc. to fund the research and, in exchange, Cepheid Inc. will provide special pricing to high-burden, low-income, developing countries. The commercial price of the GeneXpert four-module instrument is $78,200 USD, but a 78% discounted price (~$17,000 USD) was made available to developing countries [88]. In addition to FIND’s efforts, the WHO, the Bill and Melinda Gates Foundation, and the U.S. government (United States Agency for International Development and the President’s Emergency Plan for AIDS Relief) negotiated a discounted price for the test kits at $9.98 per test compared to a normal price of >$75.00 per test [88]. For new technologies to be used in low resource, point of care settings, there is a need for academic, private, and public relationship to enhance research and develop mechanisms to ensure transparency and fair pricing/reimbursement based on the ‘costs of goods sold’. Although not discussed in this review, there are rapid and low-cost antigen-based tests that detect proteins of resistance genes for isolated bacterial colonies (e.g., methicillin-resistant *S. aureus* and carbapenem-resistant Enterobacterales and *Pseudomonas aeruginosa*), which may be more cost-effective for point of care, low-resource settings [89,90,91,92,93,94].

## 8. The Future of Rapid AST

Phenotypic AST platforms currently pending regulatory approval in the United States and/or Europe include those utilizing microfluidic technologies to evaluate the electrical properties of single bacteria (iFAST Diagnostics, Northampton, UK) for both blood and urine specimens within hours and a microplate-based methodology to perform AST within 30 min (MicroplateDX, Glasgow, UK). Accelerate Diagnostics Inc., the creator of the PhenoTest is making their second-generation system, the Accelerate Wave, which will predict susceptibility using microscopy analysis (e.g., hologram). Pattern Bioscience Digital Culture (Austin, TX, USA), who received the US FDA Breakthrough Device Designation, is opting to use machine learning and single cell analysis to provide pathogen identification and phenotypic antibiotic AST.

While not novel in terms of technology, OpGen (Rockville, MD, USA) is developing the Unyvero Urinary Tract Infection (UTI) panel, which can perform identification of the most likely causative organisms in urine and identify respective AMR genes. Unlike the multiplex PCR panels that are currently on the market, the Unyvero UTI panel will include AMR genes conferring resistance to fluoroquinolones (*qnrB* and *qnrS*) and sulfonamide (*sul1*).

There are currently a variety of methods for rapid AST in the research and development phase [95]. The coupling of microfluidics and imaging has been shown to be useful in AST. Although the dRAST (QuantaMatrix, Seoul, South Korea) test uses a similar approach, there have been attempts to modify the concept to make this methodology higher throughput. Briefly, a microfluidic agarose channel (MAC) system is used to immobilize bacterial cells in agarose, and then antibiotics are allowed to permeate the system to achieve the desired concentration. Time-lapsed imaging is then able to visualize the total area of the cells that is occupied over time, as well as the morphology and growth rate, to determine antibiotic susceptibility. Single-cell morphological analysis (SCMA) was developed based on this method, which combines a MAC system chip into each well of a 96-well plate, allowing multiple antibiotic concentrations to be tested simultaneously [34,96]. However, this method requires further optimization. The main issue is the miniscule field of view (0.2mm × 0.2mm), allowing only a small number of cells to be evaluated at one time. As a result of this, it requires high bacterial loads for accurate detection. Ensuring that the technology can accommodate different bacterial morphologies, sizes, and ability to quantify bacteria in liquid phase are additional challenges.

Machine/deep learning-assisted rapid AST techniques are also in the pipeline [97]. There are efforts underway to train machine learning algorithms to appreciate the nuanced movements of bacteria in liquid culture in a way that current 2D image-processing cannot achieve [98]. As these movements change after the addition of antibiotics, it is the goal of the algorithms to appreciate this change and correlate these large clinical datasets to ultimately predict AST results. The most apparent concern with this method is that it is vulnerable to novel bacterial species and morphologies that the machine has not previously seen.

We hypothesize that we will soon see the adaptation of novel methodologies (including more artificial intelligence, next-generation sequencing) to perform rapid AST. For more details regarding specific technologies in the pipeline, please refer to a thorough review written by van Belkum et al. [95]. Currently, we mostly see rapid AST technologies validated for positive blood cultures but, in the future, additional specimen types (e.g., urine, sterile specimen types such as cerebrospinal fluid) or the inclusion of cultured bacterial isolates as a validated sample type on these commercially available platforms may be available. With increasing antimicrobial resistance, more AMR genes or drug-bug combinations may be included, such as those released by the WHO in 2024 as top priorities (e.g., cephalosporin- and/or fluoroquinolone-resistant *Neisseria gonorrhoeae*, macrolide-resistant *Streptococcus* species, ampicillin-resistant *Haemophilus influenzae*, penicillin-resistant *Streptococcus agalactiae*, macrolide-resistant *Mycoplasma genitalium*). The technologies may become cheaper, allowing them to be more financially feasible for laboratories to implement. The potential of implementing rapid AST approaches in point of care settings is enticing but the current regulatory status of these tests may hinder implementation in point of care settings. Tests used in point of care settings typically need to have a regulatory designation of ‘low risk’, ‘low complexity’, and/or ‘CLIA-waived’ status. While none of the tests described here currently have a waiver status, we hope that manufacturers will consider developing tests that fit these criteria. As medical institutions begin to appreciate and require active antimicrobial and diagnostic stewardship, we may begin to see more implementation science studies describing how medical centers can develop a multi-disciplinary team to truly maximize the utility of rapid AST technologies.

## 9. Conclusions

Conventional AST approaches typically require the growth of a pure bacterial isolate, with AST results to follow 18–24 h later. With increasing AMR, there is a clinical need for rapid AST technologies. Decreasing turnaround time to actionable AST results by days can potentially improve patient outcomes, reduce the length of admission, hospital costs, and improve antimicrobial stewardship. There are many commercially available options for rapid phenotypic and genotypic AST technologies already FDA-cleared or CE-IVD-approved for use in clinical microbiology laboratories. However, implementation is challenged by technical complexities, limitations in pathogen detection, significant costs, and the need for substantial operational adjustments. Addressing these issues requires strategic planning, thorough justification of their clinical and economic benefits, and careful management of the integration process within healthcare systems. Continuous efforts in diagnostic research and development, implementation science, and clinical outcome studies will be instrumental to improving patient care and preventing the spread of antimicrobial resistance.

## Figures and Tables

**Table 1:** Rapid phenotypic antimicrobial susceptibility testing methods approved for clinical testing.

Test [Manufactuer]	Specimen	Functionality	Organisms	Technology	Run Time per Test	Performance	Regulatory Status	Comments
PhenoTest BC [Accelerate Diagnostics Inc., (Tucson, AZ, USA)]	Blood cultures	ID and AST	GP and GN	Morphokinetic cellular analysis and fluorescence in situ hybridization	Identification in 2 h, AST in 7 h	CA: 92–99% EA: 82–97% Higher accuracy for Enterobacterales	FDA, CE-IVD	
LifeScale [Affinity Biosensors (Santa, Barbara, CA, USA)]	Blood cultures	AST only	GN	Microfluidic sensor and resonant frequency to determine organism concentration and mass distribution (e.g., growth-independent)	5 h	CA: >93.1% EA: >95.3%	FDA, CE-IVD	
ASTar [Q-linea (Uppsala, Sweden)]	Blood cultures	AST only	GN	Time-lapse imaging of bacterial growth	6 h	CA: 95–97% EA: 90–98% Lower performance in amoxicillin/ clavulanic acid and piperacillin/ tazobactam testing	FDA, CE-IVD	
VITEK REVEAL [bioMerieux (Mountain view, CA, USA)]	Blood cultures	AST only	GN	Colorimetric sensors reacting to volatile organic compounds due to bacterial metabolism during growth	5 h	CA: >96.3% EA: >98.0%	FDA, CE-IVD	1. Real-time monitoring of MICs
Selux Next-Generation Phenotyping (NGP) Test [SeluxDX (Boston, MA, USA)]	Blood cultures and bacterial colonies	AST only	GP (isolates) and GN (isolates and blood)	Fluorescent growth indictor using a viability and surface-binding assay	6–7 h	CA: >95% EA: >95%	FDA, CE-IVD	1. Requires several instruments (e.g., Separator, Inoculator, and Analyzer) 2. Can be modified into high-throughput workflow in 384-well format 3. Multiple growth control wells
QuickMIC [Gradientech (Uppsala, Sweden)]	Blood cultures	AST only	GN	Microscopic analysis of a microfluidic device	2–4 h	CA: 78–100% EA: 45–100% Low performance for tigecycline Lower performance in non-fermenters	CE-IVD	1. Determination of precise MIC values (not in doubling dilutions)
Alfred [Alifax (Padova, Italy)]	Blood cultures	AST only	GP and GN	Light scattering to detect bacterial growth	4–7 h	CA: >94% GN organisms perform slightly better than GP Lower performance in piperacillin-tazobactam testing	CE-IVD	1. MIC determination is not available
dRAST [QuantaMatrix (Seoul, South Korea)]	Blood cultures	AST only	GP and GN	Time-lapse microscopic imaging of bacterial cells	4–7 h	CA: 91–92% EA: >95% VME: 1.45–2% ME: 2.7–3.5% Minor errors: 4.8–6.6% Lower performance in gentamicin, gentamicin, piperacillin-tazobactam and cefoxitin/oxacillin testing	CE-IVD	1. Inclusion of ESBL detection
FASTinov [FASTinov (Porto, Portugal)]	Blood cultures	AST only	GP and GN	Flow cytometry using fluorescent dyes to reveal cell damage and metabolic changes (e.g., growth-independent)	2 h	CA: >96%	CE-IVD	1. Requires flow cytometry instrumentation 2. Bacterial suspension for sample preparation can be repurposed for bacterial identification (off-label) 3. Inclusion of colistin testing 4. Inclusion of ESBL detection and AmpC plasmid screening

Abbreviations: ID, identification; AST, antimicrobial susceptibility testing; h, hours; GP, Gram-positive; GN, Gram-negative; CA, categorical agreement; EA, essential agreement; VME, very major errors; ME, major errors; FDA, United States Food and Drug Administration clearance; CE-IVD, European Conformite-Europeenne in vitro Diagnostic approval; MIC, minimal inhibitory concentration; ESBL, extended spectrum beta-lactamase.

**Table 2:** Rapid genotypic antimicrobial susceptibility testing methods approved for clinical testing in Gram-positive organisms.

Test	Manufacturer	Technology	Run Time	Specimen Type	Organism	Resistance Markers		
						Methicillin	Vancomycin	Rifampin
Xpert MRSA/SA Blood Culture Assay	Cepheid (Sunnyvale, CA, USA)	PCR	1 h	Blood cultures	*Staphylococcus* species/*S. aureus*	*mecA, SCCmec/attB*	n/a	n/a
mecA XpressFish	AdvanDx (Woburn, MA, USA)	Fluorescence in situ hybridization	1 h	Blood cultures	*Staphylococcus* species/*S. aureus*	*mecA*	n/a	n/a
BD GeneOhM StaphSR Assay	Becton, Dickinson and Company (Sparks, MD, USA)	PCR	2 h	Blood cultures	*Staphylococcus* species/*S. aureus*	*SCCmec/orfX*	n/a	n/a
Great Basin Staph ID/R Blood Culture Panel	Great Basin Scientific, Inc., (West Valley City, UT, USA)	PCR	1.5 h	Blood cultures	*Staphylococcus* species/*S. aureus*	*mecA*	n/a	n/a
Xpert MRSA/SA Nasal Complete Assay	Cepheid (Sunnyvale, CA, USA)	PCR	1 h	Nasal swabs (infection control)	*Staphylococcus* species/*S. aureus*	*mecA, SCCmec/attB*	n/a	n/a
LightCycler MRSA Advanced Test	Roche, Pleasanton, CA, USA	PCR	1.5 h	Nasal swabs (infection control)	*Staphylococcus* species/*S. aureus*	*SCCmec/orfX + MREJ*	n/a	n/a
Xpert MRSA	Cepheid (Sunnyvale, CA, USA)	PCR	1 h	Nasal swabs (infection control)	*Staphylococcus* species/*S. aureus*	*mecA, SCCmec/attB*	n/a	n/a
BD GeneOhm MRSA Assay, formerly IDI-MRSA	Becton, Dickinson and Company (Sparks, MD, USA)	PCR	2 h	Nasal swabs (infection control)	*Staphylococcus* species/*S. aureus*	*SCCmec/orfX*	n/a	n/a
COBAS MRSA/SA Test	Roche (Pleasanton, CA, USA)	PCR	2 h	Nasal swabs (infection control)	*Staphylococcus* species/*S. aureus*	*SCCmec/orfX + MREJ*	n/a	n/a
Xpert MRSA NxG	Cepheid (Sunnyvale, CA, USA)	PCR	45 min	Nasal swabs (infection control)	*Staphylococcus* species/*S. aureus*	*mecA/C*, *SCCmec/orfX*	n/a	n/a
BD Max MRSA Assay	Becton, Dickinson and Company (Sparks, MD, USA)	PCR	<2 h	Nasal swabs (infection control)	*Staphylococcus* species/*S. aureus*	*SCCmec/orfX*	n/a	n/a
MRSA/SA ELITe MBG	EliTechGroup Epoech Biosciences (Paris, Ile-de-France, France)	PCR	2.5 h	Nasal swabs (infection control)	*Staphylococcus* species/*S. aureus*	*mecA*	n/a	n/a
NucliSENS EasyQ MRSA Assay	bioMerieux (Marcy-l’Étoile, France)	PCR	3 h	Nasal swabs (infection control)	*Staphylococcus* species/*S. aureus*	*Sccmec* junction and *mecA* gene	n/a	n/a
BD GeneOhm MRSA ACP Assay	Becton, Dickinson and Company (Sparks, MD, USA)	PCR	<2 h	Nasal swabs (infection control)	*Staphylococcus* species/*S. aureus*	*SCCmec/orfX*	n/a	n/a
BD GeneOhm Van R Assay	Becton, Dickinson and Company (Sparks, MD, USA)	PCR	<2 h	Peri-anal and rectal swabs (infection control)	*Enterococcus* species	n/a	*vanA/B*	n/a
IMDx Van R for Abbott m2000	Intelligent Medical Devices, Inc., (Waltham, MA, USA)	PCR	3–4 h	Peri-rectal and rectal swabs, stool (infection control)	*Enterococcus* species	n/a	*vanA/B*	n/a
Xpert vanA Assay	Cepheid (Sunnyvale, CA, USA)	PCR	45 min	Rectal swabs (infection control)	*Enterococcus* species	n/a	*vanA*	n/a
Xpert MRSA/SA SSTI Assay	Cepheid (Sunnyvale, CA, USA)	PCR	1 h	Skin and soft tissue swabs	*Staphylococcus* species/*S. aureus*	*mecA, SCCmec/attB*	n/a	n/a
Xpert MTB/RIF (Ultra) Assay	Cepheid (Sunnyvale, CA, USA)	PCR	2 h	Sputum	*Mycobacterium tuberculosis*	n/a	n/a	*rpoB*
Acuitas AMR Gene Panel	OpGen, Inc. (Rockville, MD, USA)	PCR	2.5 h	Bacterial colonies	*Enterococcus faecalis*	n/a	*vanA*	*n/a*

Abbreviations: mins, minutes; h, hours; PCR, polymerase chain reaction.

**Table 3:** Rapid genotypic AST methods approved for clinical testing in Gram-negative organisms.

Test	Manufacturer	Technology	Run Time	Specimen Type	Organism	Resistance Markers						
						ΒLac	ESBL	CARBA	AMIN	FLQ	COL	SUL
Revogene Carba C (formerly GenePOC Carba assay)	Meridian Bioscience (Cincinnati, OH, USA)	PCR	70 mins	Bacterial colonies (infection control)	Enterobacterales, *P. aeruginosa*, *A. baumannii*	n/a	n/a	*bla*IMP, *bla*NDM, *bla*VIM, *bla*OXA-48-like	n/a	n/a	n/a	n/a
Acuitas AMR Gene Panel	OpGen, Inc. (Rockville, MD, USA)	PCR	2.5 h	Bacterial colonies	Enterobacterales, *P. aeruginosa*	*blaCMY, DHA*	*blaOXA1*, *bla*OXA-9, *bla*CTX-M-1, *bla*CTX-M-2, *bla*CTX-M-9, *bla*TEM, *bla*SHV, blaPER, VEB	*blaKPC, bla*IMP, *bla*NDM, *blaVIM*, *bla*OXA-48	*aac,* *aad,* *ant,* *aph,* *armA, RMT*	*gyrA*	*mcr-1*	*Sulf1, Sulf2, DFR*
Xpert Carba-R (GNR)	Cepheid (Sunnyvale, CA, USA)	PCR	50 mins	Peri-rectal and rectal swabs, bacterial colonies (infection control)	Enterobacterales, *P. aeruginosa*, *A. baumannii*	n/a	n/a	*bla*KPC, *bla*IMP, *bla*NDM, *bla*VIM, *bla*OXA-48	n/a	n/a	n/a	n/a

Abbreviations: Blac, Beta-lactamase family; ESBL, Extended-Spectrum β-Lactamase family; CARBA, carbapenems; AMIN, aminoglycosides; FLQ, fluoronquinolones; COL, colistin; SUL, sulfonamide; mins, minutes; h, hours.

**Table 4:** Rapid genotypic syndromic panels with antimicrobial resistance markers.

Test	Manufacturer	Technology	Run Time	Specimen Type	Organism	Resistance Markers				
						ESBL	CARBA	COL	MET	VAN
ePlex Blood Culture Identification Gram Negative Panel	GenMark Diagnostics (Carlsbad, CA, USA)	PCR	1.5 h	Blood cultures	GN	*bla*CTX-M	*bla*KPC, *bla*IMP, *bla*NDM, *bla*OXA-23 blaOXA-48, *bla* VIM	n/a	n/a	n/a
ePlex Blood Culture Identification Gram Positive Panel	GenMark Diagnostics (Carlsbad, CA, USA)	PCR	1.5 h	Blood cultures	GP	n/a	n/a	n/a	*mecA/C*	*vanA/B*
BioFire FilmArray Blood Culture Identification Panel	bioMerieux (Marcy-l’Étoile, France)	PCR	1 h	Blood cultures	GP, GN, and yeast	n/a	*bla*KPC	n/a	*mecA/C*	*vanA/B*
BioFire FilmArray Blood Culture Identification 2	bioMerieux (Marcy-l’Étoile, France)	PCR	1 h	Blood cultures	GP, GN and, yeast	*bla*CTX-M	*blaKPC*, *bla*IMP, *bla*NDM, *bla*VIM, *bla* OXA-48-like	*mcr-1*	*mecA/C, mec A/C + MREJ*	*vanA/B*
Verigene Gram-Positive Nuclei Acid Test	DiaSorin (Saluggia, Italy)	Microarray	2.5 h	Blood cultures	GP	n/a	n/a	n/a	*mecA*	*vanA/B*
Verigene Gram-Negative Nuclei Acid Test	DiaSorin (Saluggia, Italy)	Microarray	2 h	Blood cultures	GN	*bla*CTX-M	*blaKPC*, *bla*IMP, *bla*NDM, *bla*VIM, *bla*OXA-48-like	n/a	n/a	n/a
BioFire FilmArray Pneumonia Panel	bioMerieux (Marcy-l’Étoile, France)	PCR	1 h	Sputum, endo-tracheal aspirate, BAL	GP, GN, atypical bacteria, and viruses	*bla*CTX-M	*bla*KPC, *bla*IMP, *bla*NDM, *bla*VIM, *bla*OXA-48-like,	n/a	*mecA/C, mec A/C + MREJ*	n/a
Unyvero LRT BAL Application	OpGen, Inc. (Rockville, MD, USA)	PCR	5 h	BAL	GP and GN	*bla*CTX-M, *bla*TEM,	*bla*KPC, *bla*NDM, *bla*VIM, *bla* OXA-23, blaOXA-24, blaOXA-48, blaOXA-58	n/a	*mecA*	n/a
BioFire FilmArray Joint Infection Panel	bioMerieux (Marcy-l’Étoile, France)	PCR	1 h	Synovial fluid	GP and GN	*bla*CTX-M	*blaKPC*, *bla*IMP, *bla*NDM, *bla*VIM, *bla*OXA-48-like	n/a	*mecA/C, mec A/C + MREJ*	*vanA/B*

Abbreviations: GP, Gram-positive; GN, Gram-negative; ESBL, Extended-Spectrum β-Lactamase family; CARBA, carbapenems; COL, colistin; MET, methicillin; VAN, vancomycin; h, hours; BAL, bronchoalveolar lavage fluid; PCR, polymerase chain reaction.

**Table 5:** Differences between phenotypic and genotypic antimicrobial susceptibility testing approaches.

Characteristic	Rapid Phenotypic Methods	Rapid Genotypic Methods
Principle	Evaluating growth, bacterial cellular and/or metabolic changes in the presence of antibiotics	Detecting the gene or mutation associated with antimicrobial resistance
Sample	Positive blood cultures, isolated bacterial colonies	For diagnostic purposes: Positive blood cultures, skin and soft tissue swabs, sputum, endotracheal aspirate, bronchoalveolar lavage, synovial fluid, isolated bacterial colonies For infection prevention/control and surveillance purposes: Nasal/peri-anal/rectal swabs, stool, isolated bacterial colonies
Identification of organism	Not available, need prior knowledge	Syndromic panels can provide both identification and AST
AST result	Antibiotic with interpretations (susceptible, intermediate, susceptible dose-dependent, resistant)	Genetic element ‘detected’ or ‘not detected’
MIC	Yes	No
Determining mechanism of resistance	No	Yes
Turnaround time	2–7 h	45 min–5 h
Adaptability	Easier to implement for new antibiotics on market	Harder to implement until a resistant mechanism is known for the antibiotic
Performance evaluation	Essential agreement: when the MIC result obtained with the AST system in evaluation (test method) is the same or within one doubling dilution step from the comparator (gold standard) method Categorical agreement: concordant interpretations (e.g., S, I, R) between the test and comparator method Very major errors: percentage of the isolates with very major errors divided by the total number of resistant isolates tested Major errors: percentage of the isolates having a major errors divided by the total number of susceptible isolates tested Minor errors: percentage of the isolates having a minor error divided by the total number of isolates tested	Sensitivity/PPA: 100 × [number of true positives detected ÷ (number of true positives + false negatives)] Specificity/NPA: 100 × [number of true negatives ÷ (number of true negatives + false positives)]

Abbreviations: AST, antimicrobial susceptibility testing; MIC, minimal inhibitory concentration; h, hours.

**Table 6:** Criteria for implementation of rapid antimicrobial susceptibility platforms.

Criteria	Considerations
Test	Performance characteristics
	Inclusion of antibiotics on hospital formulary
	Need for organism identification
	Availability of on-panel organisms
	Availability of on-panel antimicrobial genes
	Singleplex versus multiplex targets
	Number of ‘drug-bug’ combinations
	Flexibility to add more antibiotics or extend the concentrations of antibiotics tested
	Breakpoint interpretations used
	Automation versus manual hands-on time needed
Laboratory requirements	Availability of supplies and consumables
	Space requirements
	Scale of throughput
	Integration into laboratory workflow
	Cost and reimbursement
	Difficulty and expertise needed
	Maintenance needs
Clinical impact	Turnaround time
	Reporting structure changes
	Impact on patient outcomes
	Clinical education needed
	Integration with active antimicrobial stewardship

## Supplementary Materials

The following supporting information can be downloaded at: https://www.mdpi.com/article/10.3390/antibiotics13080786/s1, Table S1: Reference List of FDA-cleared rapid AST technologies and corresponding US FDA documentation.

## References

[1] Collaborators A.R. (2022). Global burden of bacterial antimicrobial resistance in 2019: A systematic analysis. Lancet.

[2] Boccabella L., Palma E.G., Abenavoli L., Scarlata G.G.M., Boni M., Ianiro G., Santori P., Tack J.F., Scarpellini E. (2024). Post-Coronavirus Disease 2019 Pandemic Antimicrobial Resistance. Antibiotics.

[3] Larkin H. (2023). Increasing Antimicrobial Resistance Poses Global Threat, WHO Says. JAMA.

[4] Balouiri M., Sadiki M., Ibnsouda S.K. (2016). Methods for in vitro evaluating antimicrobial activity: A review. J. Pharm. Anal..

[5] Ferrer R., Martin-Loeches I., Phillips G., Osborn T.M., Townsend S., Dellinger R.P., Artigas A., Schorr C., Levy M.M. (2014). Empiric antibiotic treatment reduces mortality in severe sepsis and septic shock from the first hour: Results from a guideline-based performance improvement program. Crit. Care Med..

[6] Brennan-Krohn T., Smith K.P., Kirby J.E. (2017). The Poisoned Well: Enhancing the Predictive Value of Antimicrobial Susceptibility Testing in the Era of Multidrug Resistance. J. Clin. Microbiol..

[7] Clinical Laboratory and Standards Institute (2024). Performance Standards for Antimicrobial Susceptibility Testing, CLSI Guideline M1000.

[8] United States Food and Drug Administration Antibacterial Susceptibility Test Interpretive Criteria. https://www.fda.gov/drugs/development-resources/antibacterial-susceptibility-test-interpretive-criteria.

[9] European Committee on Antimicrobial Susceptibility Testing Clinical Breakpoints—Breakpoints and Guidance. https://www.eucast.org/clinical_breakpoints.

[10] Jorgensen J.H., Ferraro M.J. (2009). Antimicrobial susceptibility testing: A review of general principles and contemporary practices. Clin. Infect. Dis..

[11] Clark R.B., Lewinski M.A., Loeffelholz M.J., Tibbetts R.J. (2009). Cumitech 31A: Verification and Validation of Procedures in the Clinical Microbiology Laboratory.

[12] Cenci E., Paggi R., Socio G.V., Bozza S., Camilloni B., Pietrella D., Mencacci A. (2020). Accelerate Pheno™ blood culture detection system: A literature review. Future Microbiol..

[13] Ullberg M., Özenci V. (2020). Identification and antimicrobial susceptibility testing of Gram-positive and Gram-negative bacteria from positive blood cultures using the Accelerate Pheno™ system. Eur. J. Clin. Microbiol. Infect. Dis..

[14] Marschal M., Bachmaier J., Autenrieth I., Oberhettinger P., Willmann M., Peter S. (2017). Evaluation of the Accelerate Pheno System for Fast Identification and Antimicrobial Susceptibility Testing from Positive Blood Cultures in Bloodstream Infections Caused by Gram-Negative Pathogens. J. Clin. Microbiol..

[15] Lutgring J.D., Bittencourt C., McElvania TeKippe E., Cavuoti D., Hollaway R., Burd E.M. (2018). Evaluation of the Accelerate Pheno System: Results from Two Academic Medical Centers. J. Clin. Microbiol..

[16] Elliott G., Malczynski M., Barr V.O., Aljefri D., Martin D., Sutton S., Zembower T.R., Postelnick M., Qi C. (2019). Evaluation of the impact of the Accelerate Pheno™ system on time to result for differing antimicrobial stewardship intervention models in patients with gram-negative bloodstream infections. BMC Infect. Dis..

[17] Starr K.F., Robinson D.C., Hazen K.C. (2019). Performance of the Accelerate Diagnostics Pheno(TM) system with resin-containing BacT/ALERT® Plus blood culture bottles. Diagn. Microbiol. Infect. Dis..

[18] De Angelis G., Posteraro B., Menchinelli G., Liotti F.M., Spanu T., Sanguinetti M. (2019). Antimicrobial susceptibility testing of pathogens isolated from blood culture: A performance comparison of Accelerate Pheno™ and VITEK® 2 systems with the broth microdilution method. J. Antimicrob. Chemother..

[19] Burg T.P., Godin M., Knudsen S.M., Shen W., Carlson G., Foster J.S., Babcock K., Manalis S.R. (2007). Weighing of biomolecules, single cells and single nanoparticles in fluid. Nature.

[20] Montelongo-Jauregui D., Slechta E.S., Fisher M. (2023). Evaluation of the LifeScale system for rapid phenotypic antimicrobial susceptibility testing from positive blood cultures. Am. J. Clin. Pathol..

[21] Banchini I., Borgatti E.C., Foschi C., Lazzarotto T., Ambretti S. (2024). Evaluation of an automated rapid phenotypic antimicrobial susceptibility testing (ASTar, Q-linea AB) applied directly on blood cultures bottles positive for Gram-negative pathogens. New Microbiol..

[22] Göransson J., Sundqvist M., Ghaderi E., Lisby J.G., Molin Y., Eriksson E., Carlsson S., Cederlöf A., Ellis L., Melin J. (2023). Performance of a System for Rapid Phenotypic Antimicrobial Susceptibility Testing of Gram-Negative Bacteria Directly from Positive Blood Culture Bottles. J. Clin. Microbiol..

[23] Esse J., Träger J., Valenza G., Bogdan C., Held J. (2023). Rapid phenotypic antimicrobial susceptibility testing of Gram-negative rods directly from positive blood cultures using the novel Q-linea ASTar system. J. Clin. Microbiol..

[24] Tibbetts R., George S., Burwell R., Rajeev L., Rhodes P.A., Singh P., Samuel L. (2022). Performance of the Reveal Rapid Antibiotic Susceptibility Testing System on Gram-Negative Blood Cultures at a Large Urban Hospital. J. Clin. Microbiol..

[25] Flentie K., Spears B.R., Chen F., Purmort N.B., DaPonte K., Viveiros E., Phelan N., Krebill C., Flyer A.N., Hooper D.C. (2019). Microplate-based surface area assay for rapid phenotypic antibiotic susceptibility testing. Sci. Rep..

[26] Baker K.R., Flentie K., Spears B.R., Mozharov S., Roberts K., El Ganbour A., Somers M., Calkwood J., Liu J., DaPonte K. (2024). Multicenter evaluation of the Selux Next-Generation Phenotyping antimicrobial susceptibility testing system. J. Clin. Microbiol..

[27] Wistrand-Yuen P., Malmberg C., Fatsis-Kavalopoulos N., Lübke M., Tängdén T., Kreuger J. (2020). A Multiplex Fluidic Chip for Rapid Phenotypic Antibiotic Susceptibility Testing. mBio.

[28] Malmberg C., Torpner J., Fernberg J., Öhrn H., Ångström J., Johansson C., Tängdén T., Kreuger J. (2022). Evaluation of the Speed, Accuracy and Precision of the QuickMIC Rapid Antibiotic Susceptibility Testing Assay with Gram-Negative Bacteria in a Clinical Setting. Front. Cell. Infect. Microbiol..

[29] Boland L., Streel C., De Wolf H., Rodriguez H., Verroken A. (2019). Rapid antimicrobial susceptibility testing on positive blood cultures through an innovative light scattering technology: Performances and turnaround time evaluation. BMC Infect. Dis..

[30] Cupaiolo R., Cherkaoui S., Serrano G., Dauby N., Georgala A., Blumental S., Maillart E., Hites M., Hallin M., Martiny D. (2022). Antimicrobial susceptibility testing determined by Alfred 60/AST (Alifax®) in a multi-sites lab: Performance’s evaluation and optimization of workflow. J. Microbiol. Methods.

[31] Anton-Vazquez V., Adjepong S., Suarez C., Planche T. (2019). Evaluation of a new Rapid Antimicrobial Susceptibility system for Gram-negative and Gram-positive bloodstream infections: Speed and accuracy of Alfred 60AST. BMC Microbiol..

[32] Sánchez-Carrillo C., Pescador P., Ricote R., Fuentes J., Losada C., Candela A., Cercenado E. (2019). Evaluation of the Alfred AST® system for rapid antimicrobial susceptibility testing directly from positive blood cultures. Eur. J. Clin. Microbiol. Infect. Dis..

[33] Giordano C., Piccoli E., Brucculeri V., Barnini S. (2018). A Prospective Evaluation of Two Rapid Phenotypical Antimicrobial Susceptibility Technologies for the Diagnostic Stewardship of Sepsis. BioMed Res. Int..

[34] Choi J., Jeong H.Y., Lee G.Y., Han S., Han S., Jin B., Lim T., Kim S., Kim D.Y., Kim H.C. (2017). Direct, rapid antimicrobial susceptibility test from positive blood cultures based on microscopic imaging analysis. Sci. Rep..

[35] Wong A.Y.W., Johnsson A.T.A., Özenci V. (2022). Performance of dRAST on Prospective Clinical Blood Culture Samples in a Simulated Clinical Setting and on Multidrug-Resistant Bacteria. Microbiol. Spectr..

[36] Kim T.Y., Kang M., Shim H.J., Kang O.K., Huh H.J., Lee N.Y. (2024). Evaluation of the QMAC-dRAST System Version 2.5 for Rapid Antimicrobial Susceptibility Testing of Gram-Negative Bacteria from Positive Blood Culture Broth and Subcultured Colony Isolates. J. Clin. Lab. Anal..

[37] Rosselin M., Prod’hom G., Greub G., Croxatto A. (2022). Performance Evaluation of the Quantamatrix QMAC-dRAST System for Rapid Antibiotic Susceptibility Testing Directly from Blood Cultures. Microorganisms.

[38] Pina-Vaz C., Silva-Dias A., Martins-Oliveira I., Gomes R., Perez-Viso B., Cruz S., Rodrigues A.G., Sarmento A., Cantón R. (2024). A multisite validation of a two hours antibiotic susceptibility flow cytometry assay directly from positive blood cultures. BMC Microbiol..

[39] Silva-Dias A., Pérez-Viso B., Martins-Oliveira I., Gomes R., Rodrigues A.G., Cantón R., Pina-Vaz C. (2021). Evaluation of FASTinov Ultrarapid Flow Cytometry Antimicrobial Susceptibility Testing Directly from Positive Blood Cultures. J. Clin. Microbiol..

[40] Fonseca E.S.D., Silva-Dias A., Gomes R., Martins-Oliveira I., Ramos M.H., Rodrigues A.G., Cantón R., Pina-Vaz C. (2019). Evaluation of rapid colistin susceptibility directly from positive blood cultures using a flow cytometry assay. Int. J. Antimicrob. Agents.

[41] Fonseca E.S.D., Andrade F.F., Gomes R., Silva-Dias A., Martins-Oliveira I., Pérez-Viso B., Ramos M.H., Rodrigues A.G., Cantón R., Pina-Vaz C. (2020). Ultra-rapid flow cytometry assay for colistin MIC determination in Enterobacterales, Pseudomonas aeruginosa and Acinetobacter baumannii. Clin. Microbiol. Infect..

[42] Cruz S., Abreu D., Gomes R., Martins-Oliveira I., Silva-Dias A., Perez-Viso B., Cantón R., Pina-Vaz C. (2024). An improved protocol for bacteria identification by MALDI-TOF MS directly from positive blood cultures. Eur. J. Clin. Microbiol. Infect. Dis..

[43] Åkerlund A., Jonasson E., Matuschek E., Serrander L., Sundqvist M., Kahlmeter G., the RAST Study Group (2020). EUCAST rapid antimicrobial susceptibility testing (RAST) in blood cultures: Validation in 55 European laboratories. J. Antimicrob. Chemother..

[44] Jonasson E., Matuschek E., Kahlmeter G. (2020). The EUCAST rapid disc diffusion method for antimicrobial susceptibility testing directly from positive blood culture bottles. J. Antimicrob. Chemother..

[45] Humphries R., Bobenchik A.M., Hindler J.A., Schuetz A.N. (2021). Overview of Changes to the Clinical and Laboratory Standards Institute Performance Standards for Antimicrobial Susceptibility Testing, M100, 31st Edition. J. Clin. Microbiol..

[46] Savage T.J., Rao S., Joerger J., Ozonoff A., McAdam A.J., Sandora T.J. (2021). Predictive Value of Direct Disk Diffusion Testing from Positive Blood Cultures in a Children’s Hospital and Its Utility in Antimicrobial Stewardship. J. Clin. Microbiol..

[47] Bard J.D., Lee F. (2018). Why Can’t We Just Use PCR? The Role of Genotypic versus Phenotypic Testing for Antimicrobial Resistance Testing. Clin. Microbiol. Newsl..

[48] Smith K.P., Kirby J.E. (2019). Rapid Susceptibility Testing Methods. Clin. Lab. Med..

[49] Simner P.J., Dien Bard J., Doern C., Kristie Johnson J., Westblade L., Yenokyan G., Patel R., Hanson K.E. (2023). Reporting of Antimicrobial Resistance from Blood Cultures, an Antibacterial Resistance Leadership Group Survey Summary: Resistance Marker Reporting Practices from Positive Blood Cultures. Clin. Infect. Dis..

[50] Becker K., Pagnier I., Schuhen B., Wenzelburger F., Friedrich A.W., Kipp F., Peters G., von Eiff C. (2006). Does nasal cocolonization by methicillin-resistant coagulase-negative staphylococci and methicillin-susceptible Staphylococcus aureus strains occur frequently enough to represent a risk of false-positive methicillin-resistant S. aureus determinations by molecular methods?. J. Clin. Microbiol..

[51] Okuma K., Iwakawa K., Turnidge J.D., Grubb W.B., Bell J.M., O’Brien F.G., Coombs G.W., Pearman J.W., Tenover F.C., Kapi M. (2002). Dissemination of new methicillin-resistant Staphylococcus aureus clones in the community. J. Clin. Microbiol..

[52] Jennings L., Van Deerlin V.M., Gulley M.L. (2009). Recommended principles and practices for validating clinical molecular pathology tests. Arch. Pathol. Lab. Med..

[53] Burd E.M. (2010). Validation of laboratory-developed molecular assays for infectious diseases. Clin. Microbiol. Rev..

[54] Simner P.J., Musser K.A., Mitchell K., Wise M.G., Lewis S., Yee R., Bergman Y., Good C.E., Abdelhamed A.M., Li H. (2022). Multicenter Evaluation of the Acuitas AMR Gene Panel for Detection of an Extended Panel of Antimicrobial Resistance Genes among Bacterial Isolates. J. Clin. Microbiol..

[55] Piatek A.S., Van Cleeff M., Alexander H., Coggin W.L., Rehr M., Van Kampen S., Shinnick T.M., Mukadi Y. (2013). GeneXpert for TB diagnosis: Planned and purposeful implementation. Glob. Health Sci. Pract..

[56] Cattamanchi A., Berger C.A., Shete P.B., Turyahabwe S., Joloba M., Moore D.A., Davis L.J., Katamba A. (2020). Implementation science to improve the quality of tuberculosis diagnostic services in Uganda. J. Clin. Tuberc. Other Mycobact. Dis..

[57] Brown S., Leavy J.E., Jancey J. (2021). Implementation of GeneXpert for TB Testing in Low- and Middle-Income Countries: A Systematic Review. Glob. Health Sci. Pract..

[58] Li S., Liu B., Peng M., Chen M., Yin W., Tang H., Luo Y., Hu P., Ren H. (2017). Diagnostic accuracy of Xpert MTB/RIF for tuberculosis detection in different regions with different endemic burden: A systematic review and meta-analysis. PLoS ONE.

[59] Cuong N.K., Ngoc N.B., Hoa N.B., Dat V.Q., Nhung N.V. (2021). GeneXpert on patients with human immunodeficiency virus and smear-negative pulmonary tuberculosis. PLoS ONE.

[60] Bouzouita I., Ghariani A., Dhaou K.B., Jemaeil S., Essaalah L., Bejaoui S., Draoui H., El Marzouk N., Mehiri E., Slim-Saidi L. (2024). Usefulness of Xpert MTB/RIF Ultra for rapid diagnosis of extrapulmonary tuberculosis in Tunisia. Sci. Rep..

[61] Dorman S.E., Schumacher S.G., Alland D., Nabeta P., Armstrong D.T., King B., Hall S.L., Chakravorty S., Cirillo D.M., Tukvadze N. (2018). Xpert MTB/RIF Ultra for detection of Mycobacterium tuberculosis and rifampicin resistance: A prospective multicentre diagnostic accuracy study. Lancet Infect. Dis..

[62] Chakravorty S., Simmons A.M., Rowneki M., Parmar H., Cao Y., Ryan J., Banada P.P., Deshpande S., Shenai S., Gall A. (2017). The New Xpert MTB/RIF Ultra: Improving Detection of Mycobacterium tuberculosis and Resistance to Rifampin in an Assay Suitable for Point-of-Care Testing. mBio.

[63] Armstrong D.T., Fisher S., Totten M., Schwartz M., Gnanashanmugam D., Parrish N. (2022). Clinical Validation of the Xpert MTB/RIF Test for Identification of the Mycobacterium tuberculosis Complex in Acid-Fast Bacillus Smear-Positive MGIT Broth Cultures. J. Clin. Microbiol..

[64] Altun O., Almuhayawi M., Ullberg M., Ozenci V. (2013). Clinical evaluation of the FilmArray blood culture identification panel in identification of bacteria and yeasts from positive blood culture bottles. J. Clin. Microbiol..

[65] She R.C., Bender J.M. (2019). Advances in Rapid Molecular Blood Culture Diagnostics: Healthcare Impact, Laboratory Implications, and Multiplex Technologies. J. Appl. Lab. Med..

[66] Ginocchio C.C., Garcia-Mondragon C., Mauerhofer B., Rindlisbacher C. (2021). Multinational evaluation of the BioFire® FilmArray® Pneumonia plus Panel as compared to standard of care testing. Eur. J. Clin. Microbiol. Infect. Dis..

[67] Klein M., Bacher J., Barth S., Atrzadeh F., Siebenhaller K., Ferreira I., Beisken S., Posch A.E., Carroll K.C., Wunderink R.G. (2021). Multicenter Evaluation of the Unyvero Platform for Testing Bronchoalveolar Lavage Fluid. J. Clin. Microbiol..

[68] Murdoch D.R., O’Brien K.L., Scott J.A., Karron R.A., Bhat N., Driscoll A.J., Knoll M.D., Levine O.S. (2009). Breathing new life into pneumonia diagnostics. J. Clin. Microbiol..

[69] Esteban J., Salar-Vidal L., Schmitt B.H., Waggoner A., Laurent F., Abad L., Bauer T.W., Mazariegos I., Balada-Llasat J.-M., Horn J. (2023). Multicenter evaluation of the BIOFIRE Joint Infection Panel for the detection of bacteria, yeast, and AMR genes in synovial fluid samples. J. Clin. Microbiol..

[70] Banerjee R., Patel R. (2023). Molecular diagnostics for genotypic detection of antibiotic resistance: Current landscape and future directions. JAC-Antimicrob. Resist..

[71] Yee R., Dien Bard J., Simner P.J. (2021). The Genotype-to-Phenotype Dilemma: How Should Laboratories Approach Discordant Susceptibility Results?. J. Clin. Microbiol..

[72] Tamma P.D., Sharara S.L., Pana Z.D., Amoah J., Fisher S.L., Tekle T., Doi Y., Simner P.J. (2019). Molecular Epidemiology of Ceftriaxone Non-Susceptible Enterobacterales Isolates in an Academic Medical Center in the United States. Open Forum Infect. Dis..

[73] Perez K.K., Olsen R.J., Musick W.L., Cernoch P.L., Davis J.R., Peterson L.E., Musser J.M. (2014). Integrating rapid diagnostics and antimicrobial stewardship improves outcomes in patients with antibiotic-resistant Gram-negative bacteremia. J. Infect..

[74] Banerjee R., Komarow L., Virk A., Rajapakse N., Schuetz A.N., Dylla B., Earley M., Lok J., Kohner P., Ihde S. (2021). Randomized Trial Evaluating Clinical Impact of RAPid IDentification and Susceptibility Testing for Gram-negative Bacteremia: RAPIDS-GN. Clin. Infect. Dis..

[75] Banerjee R., Teng C.B., Cunningham S.A., Ihde S.M., Steckelberg J.M., Moriarty J.P., Shah N.D., Mandrekar J.N., Patel R. (2015). Randomized Trial of Rapid Multiplex Polymerase Chain Reaction-Based Blood Culture Identification and Susceptibility Testing. Clin. Infect. Dis..

[76] Darie A.M., Khanna N., Jahn K., Osthoff M., Bassetti S., Osthoff M., Schumann D.M., Albrich W.C., Hirsch H., Brutsche M. (2022). Fast multiplex bacterial PCR of bronchoalveolar lavage for antibiotic stewardship in hospitalised patients with pneumonia at risk of Gram-negative bacterial infection (Flagship II): A multicentre, randomised controlled trial. Lancet Respir. Med..

[77] Miller M.M., Van Schooneveld T.C., Stohs E.J., Marcelin J.R., Alexander B.T., Watkins A.B., Creager H.M., Bergman S.J. (2023). Implementation of a Rapid Multiplex Polymerase Chain Reaction Pneumonia Panel and Subsequent Antibiotic De-escalation. Open Forum Infect. Dis..

[78] WHO (2018). WHO Guidelines Approved by the Guidelines Review Committee. Global Guidelines for the Prevention of Surgical Site Infection.

[79] Coia J.E., Wilson J.A., Bak A., Marsden G.L., Shimonovich M., Loveday H.P., Humphreys H., Wigglesworth N., Demirjian A., Brooks J. (2021). Joint Healthcare Infection Society (HIS) and Infection Prevention Society (IPS) guidelines for the prevention and control of meticillin-resistant Staphylococcus aureus (MRSA) in healthcare facilities. J. Hosp. Infect..

[80] Birgand G., Ruimy R., Schwarzinger M., Lolom I., Bendjelloul G., Houhou N., Armand-Lefevre L., Andremont A., Yazdanpanah Y., Lucet J.C. (2013). Rapid detection of glycopeptide-resistant enterococci: Impact on decision-making and costs. Antimicrob. Resist. Infect. Control.

[81] Zhou M., Kudinha T., Du B., Peng J., Ma X., Yang Y., Zhang G., Zhang J., Yang Q., Xu Y.C. (2019). Active Surveillance of Carbapenemase-Producing Organisms (CPO) Colonization with Xpert Carba-R Assay Plus Positive Patient Isolation Proves to Be Effective in CPO Containment. Front. Cell. Infect. Microbiol..

[82] Wolfe K.H., Pierce V.M., Humphries R.M. (2024). How New Regulation of Laboratory-Developed Antimicrobial Susceptibility Tests Will Affect Infectious Diseases Clinical Practice. Clin. Infect. Dis..

[83] Elnifro E.M., Ashshi A.M., Cooper R.J., Klapper P.E. (2000). Multiplex PCR: Optimization and application in diagnostic virology. Clin. Microbiol. Rev..

[84] Truong T.T., Mongkolrattanothai K., Flores I.I., Dien Bard J. (2022). Evaluation of the Performance and Clinical Impact of a Rapid Phenotypic Susceptibility Testing Method Directly from Positive Blood Culture at a Pediatric Hospital. J. Clin. Microbiol..

[85] Patel Y.A., Kirn T.J., Weinstein M.P., Uprety P. (2021). Systematic Evaluation of the Accelerate Pheno System for Susceptibility Testing of Gram-Negative Bacteria Isolated from Blood Cultures. Microbiol. Spectr..

[86] Patel J.B., Alby K., Humphries R., Weinstein M., Lutgring J.D., Naccache S.N., Simner P.J. (2023). Updating breakpoints in the United States: A summary from the ASM Clinical Microbiology Open 2022. J. Clin. Microbiol..

[87] Donner L.M., Campbell W.S., Lyden E., Van Schooneveld T.C. (2017). Assessment of Rapid-Blood-Culture-Identification Result Interpretation and Antibiotic Prescribing Practices. J. Clin. Microbiol..

[88] Gotham D., McKenna L., Deborggraeve S., Madoori S., Branigan D. (2021). Public investments in the development of GeneXpert molecular diagnostic technology. PLoS ONE.

[89] Boutal H., Vogel A., Bernabeu S., Devilliers K., Creton E., Cotellon G., Plaisance M., Oueslati S., Dortet L., Jousset A. (2018). A multiplex lateral flow immunoassay for the rapid identification of NDM-, KPC-, IMP- and VIM-type and OXA-48-like carbapenemase-producing Enterobacteriaceae. J. Antimicrob. Chemother..

[90] Hopkins K.L., Meunier D., Naas T., Volland H., Woodford N. (2018). Evaluation of the NG-Test CARBA 5 multiplex immunochromatographic assay for the detection of KPC, OXA-48-like, NDM, VIM and IMP carbapenemases. J. Antimicrob. Chemother..

[91] Jenkins S., Ledeboer N.A., Westblade L.F., Burnham C.A., Faron M.L., Bergman Y., Yee R., Mesich B., Gerstbrein D., Wallace M.A. (2020). Evaluation of NG-Test CARBA 5 for Rapid Phenotypic Detection and Differentiation of Five Common Carbapenemase Families: Results of a Multicenter Clinical Evaluation. J. Clin. Microbiol..

[92] Trienski T.L., Barrett H.L., Pasquale T.R., DiPersio J.R., File T.M. (2013). Evaluation and use of a rapid Staphylococcus aureus assay by an antimicrobial stewardship program. Am. J. Health-Syst. Pharm. AJHP.

[93] Dupieux C., Trouillet-Assant S., Tasse J., Freydière A.M., Raulin O., Roure-Sobas C., Salord H., Tigaud S., Laurent F. (2016). Evaluation of a commercial immunochromatographic assay for rapid routine identification of PBP2a-positive Staphylococcus aureus and coagulase-negative staphylococci. Diagn. Microbiol. Infect. Dis..

[94] Munier C., Dupieux C., Kolenda C., Ranc A.G., Dauwalder O., Bes M., Vandenesch F., Tristan A., Laurent F. (2023). Sensitivity of the PBP2a SA Culture Colony Test on shortly incubated subcultures of methicillin-resistant staphylococci from positive blood cultures. Diagn. Microbiol. Infect. Dis..

[95] van Belkum A., Burnham C.D., Rossen J.W.A., Mallard F., Rochas O., Dunne W.M. (2020). Innovative and rapid antimicrobial susceptibility testing systems. Nat. Rev. Microbiol..

[96] Choi J., Yoo J., Lee M., Kim E.G., Lee J.S., Lee S., Joo S., Song S.H., Kim E.C., Lee J.C. (2014). A rapid antimicrobial susceptibility test based on single-cell morphological analysis. Sci. Transl. Med..

[97] Yu H., Jing W., Iriya R., Yang Y., Syal K., Mo M., Grys T.E., Haydel S.E., Wang S., Tao N. (2018). Phenotypic Antimicrobial Susceptibility Testing with Deep Learning Video Microscopy. Anal. Chem..

[98] Frymier P.D., Ford R.M., Berg H.C., Cummings P.T. (1995). Three-dimensional tracking of motile bacteria near a solid planar surface. Proc. Natl. Acad. Sci. USA.

