# Supplementary material for: Rapid Phenotypic and Genotypic Antimicrobial Susceptibility Testing Approaches for Use in the Clinical Laboratory
Source: Antibiotics (Basel). 2024 Aug 22;13(8):786. doi: 10.3390/antibiotics13080786 (PMC11351821; doi:10.3390/antibiotics13080786)
Supplement: Supplementary file 1 [file antibiotics-13-00786-s001.zip › antibiotics-3132927-supplementary.pdf]

Table S1. Reference List of FDA-cleared rapid AST technologies and corresponding US FDA documentation

Test	Manufacturer	US Food and Drug Administration Documentation
Xpert MRSA/SA Blood Culture Assay	Cepheid (Sunnyvale, CA, USA)	https://www.accessdata.fda.gov/cdrh_docs/reviews/K101879.pdf, https://www.accessdata.fda.gov/cdrh_docs/reviews/K082140.pdf
mecA XpressFish	AdvanDx (Woburn, MA, USA)	https://www.accessdata.fda.gov/cdrh_docs/reviews/K140619.pdf
BD GeneOhM StaphSR Assay	Becton, Dickinson and Company (Sparks, MD, USA)	https://www.accessdata.fda.gov/cdrh_docs/reviews/K071026.pdf
Great Basin Staph ID/R Blood Culture Panel	Great Basin Scientific, Inc., (West Valley City, UT, USA)	https://www.accessdata.fda.gov/cdrh_docs/reviews/K152470.pdf,
Xpert MRSA/SA Nasal Complete Assay	Cepheid (Sunnyvale, CA, USA)	https://www.accessdata.fda.gov/cdrh_docs/reviews/K100822.pdf
LightCycler MRSA Advanced Test	Roche, Pleasanton, CA, USA	https://www.accessdata.fda.gov/cdrh_docs/reviews/K091409.pdf
Xpert MRSA	Cepheid (Sunnyvale, CA, USA)	https://www.accessdata.fda.gov/cdrh_docs/reviews/K070462.pdf
BD GeneOhm MRSA Assay, formerly IDI-MRSA	Becton, Dickinson and Company (Sparks, MD, USA)	https://www.accessdata.fda.gov/cdrh_docs/pdf4/K042357.pdf, https://www.accessdata.fda.gov/cdrh_docs/reviews/K033415.pdf
COBAS MRSA/SA Test	Roche (Pleasanton, CA, USA)	https://www.accessdata.fda.gov/cdrh_docs/reviews/K142721.pdf
Xpert MRSA NxG	Cepheid (Sunnyvale, CA, USA)	https://www.accessdata.fda.gov/cdrh_docs/reviews/K162444.pdf
BD Max MRSA Assay	Becton, Dickinson and Company (Sparks, MD, USA)	https://www.accessdata.fda.gov/cdrh_docs/reviews/K120138.pdf
MRSA/SA ELITe MBG	EliTechGroup Epoech Biosciences (Paris, Ile-de-France, France)	https://www.accessdata.fda.gov/cdrh_docs/reviews/K132468.pdf , https://www.accessdata.fda.gov/scripts/cdrh/cfdocs/cfPMN/pmn.cfm?ID=K112937
NucliSENS EasyQ MRSA Assay	bioMerieux (Marcy-l'Étoile, France)	https://www.accessdata.fda.gov/cdrh_docs/reviews/K102740.pdf
BD GeneOhm MRSA ACP Assay	Becton, Dickinson and Company (Sparks, MD, USA)	https://www.accessdata.fda.gov/cdrh_docs/reviews/K093346.pdf
BD GeneOhm Van R Assay	Becton, Dickinson and Company (Sparks, MD, USA)	https://www.accessdata.fda.gov/cdrh_docs/pdf10/K102416.pdf
IMDx Van R for Abbott m2000	Intelligent Medical Devices, Inc., (Waltham, MA, USA)	https://www.accessdata.fda.gov/cdrh_docs/reviews/K123753.pdf
Xpert vanA Assay	Cepheid (Sunnyvale, CA, USA)	https://www.accessdata.fda.gov/cdrh_docs/reviews/K092953.pdf
Xpert MRSA/SA SSTI Assay	Cepheid (Sunnyvale, CA, USA)	https://www.accessdata.fda.gov/cdrh_docs/reviews/K080837.pdf
Xpert MTB/RIF Assay	Cepheid (Sunnyvale, CA, USA)	https://www.accessdata.fda.gov/cdrh_docs/reviews/K143302.pdf

Revogene Carba C (formerly GenePOC Carba assay)	Meridian Bioscience (Cincinnati, OH, USA)	https://www.accessdata.fda.gov/cdrh_docs/reviews/K190275.pdf
Acuitas AMR Gene Panel	OpGen, Inc (Rockville, MD)	https://www.accessdata.fda.gov/cdrh_docs/reviews/K191288.pdf
Xpert Carba-R (GNR)	Cepheid (Sunnyvale, CA, USA)	https://www.accessdata.fda.gov/cdrh_docs/reviews/K152614.pdf
ePlex Blood Culture Identification Gram Negative Panel	GenMark Diagnostics (Carlsbad, CA, USA)	https://www.accessdata.fda.gov/cdrh_docs/reviews/K213236.pdf
ePlex Blood Culture Identification Gram Positive Panel	GenMark Diagnostics (Carlsbad, CA, USA)	https://www.accessdata.fda.gov/cdrh_docs/reviews/K181663.pdf
BioFire FilmArray Blood Culture Identification Panel	bioMerieux (Marcy-l'Étoile, France)	https://www.accessdata.fda.gov/cdrh_docs/reviews/K181493.pdf
BioFire FilmArray Blood Culture Identification 2	bioMerieux (Marcy-l'Étoile, France)	https://www.accessdata.fda.gov/cdrh_docs/reviews/K193519.pdf
Verigene Gram-Positive Nuclei Acid Test (BC-GP)	DiaSorin (Saluggia, Italy)	https://www.accessdata.fda.gov/cdrh_docs/reviews/K113450.pdf
Verigene Gram-Negative Nuclei Acid Test (BC-GN)	DiaSorin (Saluggia, Italy)	https://www.accessdata.fda.gov/cdrh_docs/reviews/K132843.pdf
BioFire FilmArray Pneumonia Panel	bioMerieux (Marcy-l'Étoile, France)	https://www.accessdata.fda.gov/cdrh_docs/reviews/K212727.pdf
Unyvero LRT BAL Application	OpGen, Inc (Rockville, MD)	https://www.accessdata.fda.gov/cdrh_docs/reviews/K191967.pdf
BioFire FilmArray Joint Infection Panel	bioMerieux (Marcy-l'Étoile, France)	https://www.accessdata.fda.gov/cdrh_docs/reviews/DEN200066.pdf
